# Multi‐Level Variable Selection Using a BART‐Enhanced Mixed‐Effects Framework

**DOI:** 10.1002/sim.70593

**Published:** 2026-05-18

**Authors:** Keming Zhang, Yaoyao Li, Jungang Zou, Sijian Wang, Bernadette A. Fausto, Liangyuan Hu

**Affiliations:** ^1^ Department of Biostatistics Brown University Providence Rhode Island USA; ^2^ Department of Biostatistics and Epidemiology Rutgers University Piscataway New Jersey USA; ^3^ Department of Biostatistics Columbia University New York New York USA; ^4^ Department of Statistics Rutgers University Piscataway New Jersey USA; ^5^ Center for Molecular and Behavioral Neuroscience Rutgers University Newark New Jersey USA; ^6^ Department of Health Science & Clinical Practice Thomas Jefferson University Philadelphia Pennsylvania USA

**Keywords:** Bayesian machine learning, Dirichlet distribution, Metropolis importance, near‐collinearity, permutation‐based, spike and slab prior

## Abstract

Selecting important individual‐ and cluster‐level predictors has become increasingly critical in healthcare research, where data often exhibit hierarchical structures due to collection from multiple clusters. Mixed‐effects models, which account for within‐cluster correlation and between‐cluster heterogeneity, are a natural approach for multilevel variable selection. However, currently available variable selection methods for multilevel data are predominantly based on mixed‐effects models that impose restrictive parametric assumptions, potentially limiting their utility when the underlying relationships are nonlinear or involve interactions. While nonparametric methods have shown promise for variable selection in non‐clustered data, they have been much less studied in the multilevel setting. Moreover, nonparametric methods that explicitly account for multilevel structure have largely been designed for prediction, rather than for simultaneous selection of relevant covariates at both the individual and cluster levels. To address these limitations, we propose a flexible, fully Bayesian unified framework for simultaneous variable selection of both fixed and random effects. Our framework integrates the nonparametric flexibility of Bayesian Additive Regression Trees (BART) for fixed‐effect predictor selection with a hierarchical Bayesian component that identifies random‐effect predictors via covariance decomposition and permutation strategies. To address scenarios common in multilevel data, where cluster‐level covariates are constant within clusters and can induce near‐collinearity and instability in selection, we further propose a computationally efficient two‐step procedure. This method disentangles the contributions of individual‐ and cluster‐level predictors, thereby mitigating collinearity and improving stability in variable selection. Comprehensive simulation studies demonstrate the effectiveness and robustness of our proposed methods across diverse scenarios. We further illustrate the practical utility of these approaches by applying them to a multilevel Alzheimer's disease dataset.

## 1 Introduction

Variable selection is a longstanding important statistical question. In healthcare research, it is common to gather data from multiple geographic locations. For instance, the Social Determinants of Health (SDOH) dataset encompasses a wide range of environmental factors—including conditions where people are born, live, learn, work, play, worship, and age—that significantly influence health outcomes, risks, and overall quality of life. Importantly, the impact of the same characteristic on health outcomes can vary substantially across different locations or among different population clusters. To account for this heterogeneity, a principled approach is to specify a hierarchical (mixed‐effects) model in which the fixed‐effects component captures the average effect of individual‐level covariates, while the random‐effects component allows those effects to vary across population clusters. Variable selection is then performed separately at each level: important individual‐level predictors are identified through fixed‐effects selection, whereas influential cluster‐level factors are detected by selecting the random‐effects terms whose variance components differ significantly from zero. This framework accommodates between‐cluster heterogeneity and yields a coherent set of predictors at both the individual and cluster levels. However, as the number of individual‐level covariates and cluster‐level predictors grows, effectively performing model selection and variable selection becomes increasingly challenging. Moreover, in many modern applications, interest lies not only in prediction but also in obtaining interpretable insight into the factors associated with within‐cluster outcome variation and between‐cluster heterogeneity, which further increases the demands on multilevel variable selection methods.

Numerous studies have demonstrated the advantages of flexible tree‐based machine learning methods—such as Random Forests [1], Extreme Gradient Boosting [2], and Bayesian Additive Regression Trees (BART) [3]—for fixed‐effects variable selection [4, 5, 6, 7, 8]. Tree‐based models partition the predictor space into distinct regions, enabling them to effectively capture complex interactions and nonlinear relationships between covariates and the response. Among these approaches, BART has gained considerable attention due to its robust predictive performance and accuracy [3, 5, 8, 9]. BART is a Bayesian nonparametric ensemble method that combines predictions from multiple regression trees in a sequential boosting‐like manner. Embedded within a probabilistic Bayesian framework, BART employs regularizing priors on tree parameters to constrain individual tree contributions, thus preventing overfitting while preserving high predictive accuracy. Variable importance can be effectively assessed through metrics such as variable inclusion proportions and Metropolis importance. BART has been extended to clustered and longitudinal settings by incorporating random effects. Examples include mixedBART, which combines BART with mixed‐model components to accommodate within‐cluster dependence, and related multilevel extensions such as HE‐BART [9, 10]. Our previous work on riAFT‐BART and related accelerated failure time extensions similarly integrates BART with random intercepts, although it is tailored to clustered survival outcomes [7, 11]. Collectively, these approaches demonstrate the usefulness of combining tree‐based models with random effects for flexible analysis of clustered data. However, they are aimed primarily at prediction and flexible mean modeling rather than simultaneous variable selection at multiple levels. Specifically, they do not directly address two issues that are fundamental to multilevel variable selection: principled selection of the random‐effects structure, and stable identification of cluster‐level covariates that are constant within clusters when random intercepts are included.

In contrast, variable selection for random effects has received comparatively less attention. Random predictors are considered important if their coefficients exhibit meaningful variability across clusters. Traditional model selection criteria, such as Akaike's Information Criterion (AIC) and Bayesian Information Criterion (BIC), can rank mixed‐effects models once they are fitted, but selecting a random‐effects structure may still be demanding because (i) the number of candidate structures grows rapidly, and (ii) the usual AIC/BIC penalties provide no consensus on how to account for the complexity of variance–covariance parameters. Alternative approaches have primarily employed penalized likelihood methods within parametric modeling frameworks. For instance, Bondell et al. [12] proposed a penalized joint log‐likelihood approach incorporating adaptive penalties for simultaneously selecting fixed and random effects. Peng and Yu [13] introduced a method leveraging the partial consistency property of random coefficients to identify significant random‐effect predictors. Additionally, Hui et al. [14] developed a method combining penalized quasi‐likelihood (PQL) estimation with adaptive lasso and adaptive group lasso penalties to select fixed and random coefficients, respectively. Beyond penalized likelihood methods, parametric Bayesian hierarchical approaches have also been explored. Chen and Dunson [15] proposed a hierarchical Bayesian framework for selecting random effects by decomposing the covariance matrix of random coefficients using a modified Cholesky factorization, assuming prior accurate selection of the fixed‐effect component. Related Bayesian selection ideas for mixed models and additive mixed models include spike‐and‐slab formulations that induce sparsity on fixed effects and/or structured random‐effect components [16, 17].

While BART has demonstrated considerable success in selecting fixed effects, its flexibility and predictive accuracy have not been fully utilized in multilevel variable selection. Existing multilevel variable selection methods typically rely on parametric assumptions, making them vulnerable to model misspecification—especially in complex data scenarios—resulting in incorrect predictor selection and inferential biases. To overcome these limitations, we first propose a flexible, fully Bayesian unified framework that simultaneously and efficiently selects both fixed and random effects. Our approach integrates BART for flexible individual‐level variable selection with a parametric hierarchical Bayesian component to identify random effects through covariance matrix decomposition and permutation methods. Relative to existing multilevel BART approaches (e.g., mixedBART [9] and HE‐BART [10]), the methodological novelty here is selection‐oriented: we integrate existing BART variable selection methods (permutation‐based [8] and Dirichlet prior [18]) with a random‐effect selection mechanism within a Bayesian hierarchy and provide posterior evidence for which random‐effect components are needed, rather than treating the random‐effects structure as fixed.

We then address another fundamental challenge in multilevel selection. In multilevel data, cluster‐level covariates often do not vary within clusters. When such covariates are modeled alongside random intercepts in a unified selection framework, both terms compete to explain the same between‐cluster signal, inducing near‐collinearity and destabilizing variable identification. A “random‐slope–only” specification does not resolve this: random slopes address heterogeneity in within‐cluster effects of individual‐level covariates and are not identified for covariates that are constant within clusters; moreover, slope‐only models can be fragile with small or unbalanced clusters, further undermining selection stability. This near‐collinearity issue is typically not addressed explicitly in multilevel BART implementations because it is less visible when the goal is prediction, but it becomes critical for reliable variable selection and scientific interpretation of cluster‐constant covariates. To address this challenge, we propose a two‐step selection procedure that decouples within‐ from between‐cluster signal. In Step 1, we fit a BART model to individual‐level covariates with random intercepts to absorb cluster heterogeneity. In Step 2, we regress the estimated random intercepts (e.g., posterior means) on cluster‐level covariates and apply permutation‐based selection. By separating individual‐ and cluster‐level effects, this procedure effectively mitigates the near‐collinearity induced when cluster‐constant covariates are modeled jointly with random intercepts, thereby improving the stability and accuracy of variable selection at both levels. This two‐step component is a second key novelty: it targets a pervasive identifiability/stability problem for cluster‐constant covariates that is often ignored in unified multilevel selection frameworks.

The remainder of the paper is organized as follows. In Section 2, we introduce notation and describe our proposed BART‐enhanced unified model designed for simultaneous multilevel variable selection. We further propose a two‐step approach specifically designed to mitigate the near‐collinearity that arises between random intercepts and cluster‐level predictors that remain constant within clusters. Section 3 evaluates the performance of our proposed methods through comprehensive simulations under two distinct scenarios. First, we demonstrate the efficacy of our unified model when cluster‐level variables vary within clusters. We then illustrate challenges due to near‐collinearity when these variables are constant within clusters, showing how our two‐step approach effectively resolves this issue, enabling accurate and reliable selection at both individual and cluster levels. In Section 4, we demonstrate our methods via a case study, identifying important predictors of Alzheimer's disease risk at both the individual and community levels. Section 5 summarizes our key findings, discusses their implications, highlights the strengths and limitations of our approaches, and outlines potential directions for future research.

## 2 Methodology

### 2.1 Notation

Consider a hierarchical dataset with K clusters, where the k‐th cluster contains $n_{k}$ individuals. The data include both individual‐level and cluster‐level covariates. Let X denote the design matrix of P individual‐level covariates, and let Z represent the design matrix of Q cluster‐level covariates. The first column of Z is set to 1 to accommodate a random intercept. For the j‐th variable, let $X_{j}$ and $Z_{j}$ denote the corresponding columns of X and Z, respectively, representing the values of the j‐th individual‐level and cluster‐level covariates across all observations. For i‐th individual in the k‐th cluster, denote the individual‐level and cluster‐level covariate vector as $X_{\mathrm{ik}}$ and $Z_{\mathrm{ik}}$, respectively. and let $X_{\mathrm{ikj}}$ and $Z_{\mathrm{ikj}}$ denote the j‐th covariate within vectors, respectively. Let y denote the vector of observed outcomes, and let $y_{\mathrm{ik}}$ be the response variable for the i‐th individual in the k‐th cluster.

### 2.2 Unified Model

Consider the following mixed‐effects model, which includes a (potentially nonparametric) function of the individual‐level covariates X as fixed effects and a cluster‐specific random effect:

(1) $$y_{\mathrm{ik}}=f\left(X_{\mathrm{ik}}\right)+Z_{\mathrm{ik}}\beta_{k}+\varepsilon_{\mathrm{ik}},$$

where $f\left(X_{\mathrm{ik}}\right)$ is an unknown (possibly nonparametric) function relating $X_{\mathrm{ik}}$ to $y_{\mathrm{ik}}$, $Z_{\mathrm{ik}}$ is the design matrix for the random effects, $\beta_{k}$ is the Q×1 vector of random coefficients for cluster k (including the random intercept), and $\varepsilon_{\mathrm{ik}}$ is the random error term. We assume $\beta_{k}\overset{i.i.d}{∼}ℳ\mathrm{VN}\left(0,D\right)$, where D is the Q×Q covariance matrix capturing the variances and covariances of the random effects. The residuals are assumed to be $\varepsilon_{\mathrm{ik}}\overset{i.i.d}{∼}N\left(0,\sigma^{2}\right)$ and independent of the random effects $\beta_{k}$.

We employ BART to estimate the unknown function f for the fixed‐effects. BART [3] is a Bayesian non‐parametric approach to function estimation and inference, representing a target function as the sum of H regression trees: $f\left(X_{\mathrm{ik}}\right)=\sum_{h=1}^{H}g\left(X_{\mathrm{ik}};T_{h},\mu_{h}\right)$, where $g\left(X_{\mathrm{ik}};T_{h},\mu_{h}\right)$ denotes a single Bayesian regression tree. Each tree is defined by a structure $T_{h}$, which specifies a set of interior decision nodes and their splitting rules, along with $b_{h}$ terminal nodes. These terminal nodes are parametrized by the vector $\mu_{h}=\left(\mu_{h,1},\ldots ,\mu_{h,b_{h}}\right)$. Hence, $g\left(X_{\mathrm{ik}};T_{h},\mu_{h}\right)=\mu_{h,d}$ if $X_{\mathrm{ik}}$ falls into the d‐th terminal node. By applying BART within our mixed‐effects framework, we obtain the following unified model:

(2) $$y_{\mathrm{ik}}=\sum_{h=1}^{H}g\left(X_{\mathrm{ik}};T_{h},\mu_{h}\right)+Z_{\mathrm{ik}}\beta_{k}+\varepsilon_{\mathrm{ik}},$$

This formulation captures individual‐level variation through $f\left(X_{\mathrm{ik}}\right)$ and cluster‐specific variation through $Z_{\mathrm{ik}}\beta_{k}$. To jointly estimate and simultaneously select individual‐level and cluster‐level variables in a single coherent framework, we cast the procedure in a fully Bayesian model, enabling unified uncertainty quantification and posterior‐based selection. We compute BART variable‐inclusion measures for individual‐level covariates and compare each to its permutation‐based null distribution. For cluster‐level covariates, we reparameterize the random‐effects covariance D via its Cholesky factor and apply the same permutation calibration for selection. Using permutation‐derived nulls provides explicit error control under complex dependence, yielding a coherent selection rule across levels.

#### 2.2.1 Individual‐Level Selection

We begin by illustrating the selection of individual‐level covariates in X based on our unified model (2). We consider four existing methods for evaluating variable importance in fixed‐effect selection: variable inclusion proportions (VIP), its extension VIP with Type Information, and Metropolis Importance (MI) and Dirichlet prior over the variable selection probabilities [4, 8, 18]. Each method addresses different challenges in identifying relevant covariates.

The VIP method evaluates a covariate's importance by counting the frequency of its appearance in splitting rules across the regression trees. Given M posterior samples, let $c_{\mathrm{jm}}$ denote the number of times that covariate $X_{j}$ appears in the splitting rules of the m‐th posterior sample, and the VIP score for $X_{j}$ is defined as

$$v_{j}=\frac{1}{M}\sum_{m=1}^{M}\frac{c_{\mathrm{jm}}}{\sum_{l=1}^{P}c_{\mathrm{lm}}}.$$

A higher $v_{j}$ indicates that $X_{j}$ is more relevant. However, this basic VIP measure can favor continuous covariates, because binary variables have fewer possible splits. To address this issue, *VIP with Type Information* normalizes the importance scores within each covariate type (continuous vs. binary). Let $c_{\mathrm{cts},m}$ and $c_{\mathrm{bin},m}$ denote the total number of splits for all continuous and binary covariates, respectively, in the m‐th posterior sample. The adjusted VIP score is then

$$v_{j}^{w.t.}=\frac{1}{M}\sum_{m=1}^{M}\frac{c_{\mathrm{jm}}}{c_{\mathrm{cts},m}I\left(X_{j}\;\text{is}\;\text{continuous}\right)+c_{\mathrm{bin},m}I\left(X_{j}\;\text{is}\;\text{binary}\right)},$$

thereby preventing binary variables from being overshadowed by continuous ones.

When the number of covariates is small or additional covariate types are present, VIP‐based methods may still struggle. To address this, the MI approach leverages the Metropolis‐Hastings acceptance ratio within BART's tree‐sampling process. During each tree update in BART, a leaf node is randomly selected for one of four modifications: BIRTH (splitting into two leaves), DEATH (collapsing adjacent leaves), CHANGE (reassigning a decision rule), or SWAP (swapping decision rules between nodes) [19]. Although all four moves are available, BIRTH and DEATH alone suffice to construct a valid BART model [20]. Consider a splitting node η in the h‐th tree $T_{h}$. Let $T_{h}^{*}$ be the new tree proposed by applying a BIRTH operation. The acceptance probability for this proposal is $\underset{\text{BIRTH}}{\mathrm{Pr}}\left(\eta \right)=\min\left\{1,\frac{p\left(T_{h}\;|\;T_{h}^{*}\right)p\left(T_{h}^{*}\;|-\right)}{p\left(T_{h}^{*}\;|\;T_{h}\right)p\left(T_{h}\;|-\right)}\right\}$, where $p\left(T_{h}^{*}|T_{h}\right)$ is the proposal distribution for moving from the current tree $T_{h}$ to the new tree $T_{h}^{*}$, $p\left(T_{h}|T_{h}^{*}\right)$ is the reverse proposal distribution, indicating how likely it would be to go from $T_{h}^{*}$ back to $T_{h}$, $p\left(T_{h}^{*}\;|-\right)$ is the target distribution of the proposed tree $T_{h}^{*}$, and $p\left(T_{h}\;|-\right)$ is the target distribution of the current tree $T_{h}$. The MI score for covariate $X_{j}$ is computed by first averaging its acceptance ratios across trees and posterior samples:

$${\tilde{c}}_{\mathrm{jm}}=\frac{\sum_{h=1}^{H}\sum_{\eta \in \phi_{\mathrm{hm}}}I\left(j_{\eta }=j\right)\underset{\text{BIRTH}}{\mathrm{Pr}}\left(\eta \right)}{\sum_{h=1}^{H}\sum_{\eta \in \phi_{\mathrm{hm}}}I\left(j_{\eta }=j\right)},$$

where $\phi_{\mathrm{hm}}$ is the set of split nodes in the h‐th tree of the m‐th posterior sample, and $j_{\eta }$ indicates the covariate used at node η. The MI score for $X_{j}$ is then normalized as

$$v_{j}^{\mathrm{MI}}=\frac{1}{M}\sum_{m=1}^{M}\frac{{\tilde{c}}_{\mathrm{jm}}}{\sum_{l=1}^{P}{\tilde{c}}_{\mathrm{lm}}}.$$

Whereas VIP and its type‐adjusted variant rely on how often a covariate appears in splits, MI gauges each covariate's impact through accepted tree updates, yielding a more robust selection criterion.

Unlike the three methods mentioned above that are applied within the default BART model, Linero [18] proposed an extension that places a Dirichlet prior on the variable–selection probabilities to induce sparsity. Let $s=\left(s_{1},\ldots ,s_{P}\right)$ lie on the probability simplex (i.e., $\sum_{j=1}^{P}s_{j}=1$), where $s_{j}$ is the probability that predictor j is used to construct a given split. The default BART model assumes $s_{j}=1/P$ for all j, so that predictors are chosen uniformly. Linero [18] instead uses a sparsity–inducing Dirichlet prior,

$$\left(s_{1},\ldots ,s_{P}\right)∼\text{Dirichlet}\left(\frac{\alpha }{P},\ldots ,\frac{\alpha }{P}\right),$$

where α controls the overall sparsity level (see Linero [18] for guidance on choosing α). This approach constrains splits to a sparse subset of variables, allowing the model to learn a splitting distribution and interpret higher selection probabilities as signals of greater variable importance.

#### 2.2.2 Cluster‐Level Selection

Although individual‐level selection identifies covariates that directly influence the response, hierarchical models introduce additional complexity through cluster‐level random effects. These effects, denoted by β, capture unobserved heterogeneity across clusters and can be correlated with cluster‐specific covariates Z. The next step in our approach is to identify relevant cluster‐level predictors that explain between‐cluster variation, ensuring a comprehensive understanding of multilevel data structures.

We assume $\beta_{k}\overset{i.i.d}{∼}ℳ\mathrm{VN}\left(0,D\right)$ for the k‐th cluster. The q‐th random effect is deemed insignificant if the q‐th diagonal element of D is zero, indicating no between‐cluster variability in that effect. Directly testing whether each diagonal element of D is zero, however, can be challenging. Following Chen and Dunson [15], we use the modified Cholesky decomposition to express $D={\Lambda \Gamma \Gamma }^{⊤}\Lambda ,$ where $\Lambda =\mathrm{diag}\left(\lambda_{1},\ldots ,\lambda_{Q}\right)$ is a diagonal matrix whose entries are proportional to the variances of the random effects, and Γ is a Q×Q lower triangular matrix capturing their correlations [15]. By allowing each $\lambda_{q}$ on the diagonal of Λ to be zero with positive probability, random effects with zero variance can be effectively excluded from the model. Let L=ΛΓ be the lower triangular Cholesky decomposition of D, and let $\gamma_{\mathrm{qu}}$ denote the (q,u)‐th element of Γ. To ensure Λ and Γ are uniquely identifiable for a given L, we impose the constraint: 

(3)

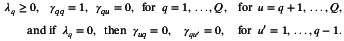




Under these constraints, Λ remains a diagonal matrix with nonnegative entries, while Γ is lower triangular with ones on the diagonal. We then reparametrize the mixed‐effects model (2) as:

(4) $$y_{\mathrm{ik}}=\sum_{h=1}^{H}g\left(X_{\mathrm{ik}};T_{h},\mu_{h}\right)+Z_{\mathrm{ik}}\Lambda \Gamma b_{k}+\varepsilon_{\mathrm{ik}},$$

where $b_{k}={\left(b_{k1},\ldots ,b_{\mathrm{kQ}}\right)}^{T}$ follows an i.i.d standard normal distribution. This reformulation simplifies the task of determining whether each diagonal element of D is zero into checking whether each $\lambda_{q}$ is zero, thereby improving the tractability of random‐effect selection in hierarchical models.

To enable a positive probability of excluding each random effect, we assign independent spike‐and‐slab priors to each $\lambda_{q}$, ensuring $\lambda_{q}=0$ with nonzero probability:

(5) $$\begin{array}{cc} \lambda_{q}\;|\;v_{q} & ∼\left(1-v_{q}\right)\delta_{0}+v_{q}\mathrm{TN}\left(\theta_{q},s_{q}^{2},\left(0,\infty \right)\right),\\ v_{q} & ∼\text{Bernoulli}\left(\pi_{q}\right),\;\text{for}\;\forall q=1,\ldots ,Q, \end{array}$$

where $\delta_{0}$ is the Dirac delta function at zero, $v_{q}$ is a binary indicator for $\lambda_{q}\neq 0$, $\theta_{q}$, and $s_{q}^{2}$ are mean and variance for the positive component of $\lambda_{q}$, and $\pi_{q}$ denotes the probability of $\lambda_{q}\neq 0$. Since $\lambda_{q}$ must be nonnegative, the standard normal component in the usual spike‐and‐slab prior is replaced with a truncated normal on (0,∞), denoted $\mathrm{TN}\left(\theta_{q},s_{q}^{2},\left(0,\infty \right)\right)$ [21]. The spike‐and‐slab prior serves two key purposes. When $v_{q}=0$, $\lambda_{q}$ is forced to be zero, effectively excluding the corresponding random effect and indicating that its associated cluster‐level covariate $Z_{q}$ is not selected. When $v_{q}=1$, $\lambda_{q}$ follows a half normal distribution, preserving the random effect. In practice, the posterior draws for each $v_{q}$ are rarely all zeros or ones, which creates ambiguity in deciding how to include or exclude the effect. To address this issue, we develop a rigorous, permutation‐based procedure for multilevel variable selection under our unified model, as detailed in Section 2.2.3.

#### 2.2.3 Permutation‐Based Variable Selection

In hierarchical models, selecting the appropriate random‐effects structure requires determining which subset of cluster‐level covariates is active. Given that the number of random predictors in healthcare research is typically small and their random‐effect coefficients often exhibit strong dependence, we consider all $2^{Q}$ candidate submodels, ranging from the null model (no random effects) to the full model (all Q random predictors). Under a Bayesian framework, each submodel ${ℳ}_{r}$ receives a posterior probability proportion to how well it explains the response, so submodels that better capture the data are selected more frequently. To measure the relevance importance of submodel ${ℳ}_{r}$, we define its Lambda Positivity Score (LPS) by

$${\mathrm{LPS}}_{r}=\frac{\text{Number of posterior draws in which submodel}\;{ℳ}_{r}\;\text{is selected}}{M},$$

where M is the total number of posterior draws. A higher ${\mathrm{LPS}}_{r}$ indicates that the variance parameters $\left\{\lambda_{q}\right\}$ associated with submodel ${ℳ}_{r}$ are more consistently nonzero, suggesting a stronger association between the corresponding cluster‐level predictors and the response.

Instead of relying on a single observed value of a variable‐importance measure, we implement a permutation‐based variable selection procedure that generates an empirical null distribution for each variable's importance score. By comparing the observed importance measures from the original (unpermuted) dataset to their respective null distributions, we enable a principled and rigorous identification of relevant variables. This permutation procedure applies to both individual‐level and cluster‐level covariates. For individual‐level covariates X, we employ three BART‐based importance metrics—VIP, Within‐Type VIP, and MI—while for random‐effects structures specified by covariates Z, we utilize the LPS as the primary selection criterion. For sparsity‐inducing BART, we employ the selection rule in Section 2.2.1.

Our permutation scheme approximates the reference (“null”) distribution in which the outcome is independent of the covariates, while respecting the hierarchical structure of Z. When Z varies within clusters, we test the joint‐independence hypothesis $H_{0}:Y⊥\left(X,Z\right)$; to generate null replicates we first randomly permute the individual‐level realizations of Z within each cluster—severing any alignment of Z with Y and X while preserving cluster membership—and then permute Y within clusters to eliminate any remaining association with the covariates. This joint‐independence formulation avoids restrictive modeling assumptions and accommodates potential correlations and interactions among covariates. When Z is constant within clusters (i.e., cluster‐level), we instead test $H_{0}:Y⊥\;X|Z$ and permute Y within clusters, disrupting any association with X while preserving the between‐cluster structure induced by Z.

Under the null hypothesis described above, we generate L permuted datasets and fit the model to each, yielding L realizations of (i) the three variable‐importance scores for each covariate $X_{j}$ and (ii) the local predictive score (LPS) for each submodel, thereby forming permutation‐based null distributions for these quantities. For a chosen significance level α, a covariate or submodel is declared significant if its observed (unpermuted) importance score or LPS exceeds the (1−α) quantile of the corresponding null distribution, indicating that selection is unlikely to be due to chance. If multiple submodels meet this criterion, we retain the one with the largest observed LPS. To improve robustness, we also run $L_{\mathrm{rep}}$ independent MCMC chains on the unpermuted data from dispersed initializations; VIP, within‐type VIP, and LPS are averaged across chains, whereas MI is aggregated by the median to limit outlier effects [8]. This procedure yields stable variable‐selection decisions that are not unduly driven by random variation. The complete algorithm is detailed in Web Algorithm 1 of the Supporting Information. The default values of L, $L_{\mathrm{rep}}$, and α are 100, 10, and 0.05, respectively.

For categorical covariates, we first encode them as sets of binary (dummy) variables before model fitting. After fitting the model, the importance metrics or LPS values of all dummy variables corresponding to the same categorical covariate are summed. The resulting value is then treated using the same rules applied to continuous covariates.

Under sparsity‐inducing BART, variable selection proceeds by shrinking each predictor's chance of being chosen for tree splits toward zero unless the data provide strong evidence to the contrary. After fitting the model, we summarize each predictor's selection frequency across trees and posterior draws, rank predictors by these probabilities, and identify a data‐driven cutoff at the largest drop (“elbow”) in the ranked sequence; predictors above this cutoff are retained as individual‐level covariates. For the selection of cluster‐level (random) effects, we choose the submodel that attains the highest LPS.

#### 2.2.4 Prior Specification

We now specify the prior distributions for each component of the unified model (4)–BART, random effects, and residuals–under the assumption that these components are mutually independent.

For the BART component, we employ an ensemble of H independent regression trees, ${\left\{T_{h}\right\}}_{h=1}^{H}$, each associated with leaf parameters ${\left\{\mu_{h}\right\}}_{h=1}^{H}$, following standard prior specifications from previous work [3, 22]. Consistent with recommendations by Bleich [4], we set H=20 to limit ensemble complexity, thereby encouraging competition among predictors and reducing the likelihood that irrelevant variables are selected. This choice is supported by stable and reliable performance observed in our simulations (Section 3). For the sparsity‐inducing BART, we follow the same prior specification in literature [18].

For the random‐effects covariance structure, we adopt a factorization of the covariance matrix D as $D={\Lambda \Gamma \Gamma }^{⊤}\Lambda$, where Λ is a diagonal matrix and Γ is a lower‐triangular matrix with ones on the diagonal. Instead of directly assigning a prior to D, we specify priors for the elements of Λ and Γ. Specifically, each diagonal element $\lambda_{q}$ of Λ follows a spike‐and‐slab prior as in (5). The inclusion probability $\pi_{q}$ for each $\lambda_{q}$ is modeled with a Beta prior, $\pi_{q}∼\text{Beta}\left(c_{q},d_{q}\right)$, where hyperparameters $c_{q}$ and $d_{q}$ are set to 0.5, corresponding to Jeffrey's prior for the Beta distribution. Conditional on Λ, the strictly lower‐triangular elements of Γ (organized into a vector γ) follow a mixture prior distribution consisting of a multivariate normal distribution, $N\left(\gamma_{0},R_{0}\right)$, and a point mass at zero. Under this mixture prior, each off‐diagonal element $\gamma_{\mathrm{uq}}$ is set to zero whenever the corresponding diagonal elements satisfy $\lambda_{u}=0$ or $\lambda_{q}=0$. Default hyperparameters are set as $\gamma_{0}=0$ and $R_{0}=0.5\;I$, where I is the identity matrix of size Q(Q−1)/2×Q(Q−1)/2.

Finally, for the residual variance $\sigma^{2}$, we adopt a scaled inverse‐$\chi^{2}$ prior:$\sigma^{2}∼\frac{\nu \;\tau^{2}}{\chi_{\nu }^{2}},$ with degrees of freedom ν=3 to ensure weakly informative behavior. The scale parameter $\tau^{2}$ is chosen using an empirical Bayes approach, matching the residual variance from an initial least‐squares fit [3].

Even though prior sensitivity is a legitimate concern in multilevel selection, our prior specification has already combined both the standard weakly informative choices that are widely used in the BART and Bayesian mixed‐effects literature, and calibrated components introduced only to place parameters on an appropriate scale. Specifically, the BART tree‐structure and leaf‐parameter priors follow the default regularization in Chipman et al. [3] and the implementation in Sparapani et al. [22], and the sparsity‐inducing Dirichlet splitting prior follows Linero [18]; these are conventional “reference” specifications designed to stabilize function estimation rather than inject strong prior beliefs. For the random‐effects covariance, the modified Cholesky parameterization [15] is paired with spike‐and‐slab priors on $\left\{\lambda_{q}\right\}$ (Equation (5)) to induce exact exclusion with positive probability; here, the *inclusion prior* $\pi_{q}∼\text{Beta}\left(0.5,0.5\right)$ is a Jeffreys‐type reference choice, while the *slab scale* $\left(\theta_{q},s_{q}^{2}\right)$ controls how strongly nonzero random‐effect variances are encouraged a priori, and it has been shown in the related paper that the model is robust with different slab prior [15]. Finally, the residual prior $\sigma^{2}∼{\nu \tau }^{2}/\chi_{\nu }^{2}$ uses a weak degrees‐of‐freedom setting (ν=3) with an empirical‐Bayes scale $\tau^{2}$ to match the observed outcome magnitude, which improves numerical stability but does not impose sparsity or favor particular predictors.

ALGORITHM 1 **One iteration of Gibbs sampling algorithm for updating the unified model.**

1: Regress $y_{\mathrm{ik}}-Z_{\mathrm{ik}}\Lambda \Gamma b_{k}$ on $X_{\mathrm{ik}}$ via Bayesian backfitting sampling algorithm to update ${\left\{T_{h},\mu_{h}\right\}}_{h=1}^{H}$, $i=1,\ldots ,n_{k}$, k=1,…,K.

2: Update $f\left(X_{\mathrm{ik}}\right)=\sum_{h=1}^{H}g\left(X_{\mathrm{ik}};T_{h},\mu_{h}\right)$, $i=1,\ldots ,n_{k}$, k=1,…,K.

3: Sample $b_{k}$ from $ℳ\mathrm{VN}\left({\hat{h}}_{k},{\hat{H}}_{k}\right)$, where ${\hat{H}}_{k}={\left(\sigma^{-2}\sum_{i=1}^{n_{k}}\Gamma^{⊤}\Lambda Z_{\mathrm{ik}}^{⊤}Z_{\mathrm{ik}}\Lambda \Gamma +I\right)}^{-1}$ and ${\hat{h}}_{k}=\sigma^{-2}\sum_{i=1}^{n_{k}}Z_{\mathrm{ik}}\Lambda \Gamma {\left(y_{\mathrm{ik}}-f\left(X_{\mathrm{ik}}\right)\right)}^{⊤}{\hat{H}}_{k}$,k=1,…,K.

4: Sample γ from $ℳ\mathrm{VN}\left(\hat{\gamma },\hat{R}\right)$, where $\hat{\gamma }=\hat{R}\left(\sigma^{-2}\sum_{k=1}^{K}\left(\sum_{i=1}^{n_{k}}{\tilde{u}}_{\mathrm{ik}}(y_{\mathrm{ik}}-f\left(X_{\mathrm{ik}}\right)-Z_{k}\Lambda b_{k}^{T}\right)+R_{0}^{-1}\gamma_{0}\right)$ and $\hat{R}={\left(\sigma^{-2}\sum_{k=1}^{K}\sum_{i=1}^{n_{k}}{\tilde{u}}_{\mathrm{ik}}{\tilde{u}}_{\mathrm{ik}}^{⊤}+R_{0}^{-1}\right)}^{-1}$, where ${\tilde{u}}_{\mathrm{ik}}=\left(b_{\mathrm{kq}}\lambda_{u}Z_{\mathrm{iku}}:q=1,\ldots ,Q;u=q+1\right)$ is a $\frac{Q\left(Q-1\right)}{2}$ dimensional vector. Transform γ back to recover Γ.

5: Sample $\pi_{q}$ from $\text{Beta}\left(c_{q}+v_{q},d_{q}+1-v_{q}\right)$, q=1,…,Q.

6: Sample $v_{q}$ from $\text{Bernoulli}\left(\frac{\pi_{q}{ℒ}_{1}}{\pi_{q}{ℒ}_{1}+\left(1-\pi_{q}\right){ℒ}_{0}}\right)$, q=1,…,Q, where ${ℒ}_{1}$ and ${ℒ}_{0}$ are defined in Web Formula (2) in Supporting Information, respectively.

7: Set $\lambda_{q}=0$ if $v_{q}=0$, q=1,…,Q.

8: Sample $\lambda_{q}$ from TN(${\hat{\lambda }}_{q}$, ${\hat{s}}_{q}^{2}$, (0, ∞)), if $v_{q}=1$, q=1,…,Q, where ${\hat{\lambda }}_{q}$ and ${\hat{s}}_{q}^{2}$ are defined in Web Formula (1) in Supporting Information, respectively.

9: Sample $\sigma^{2}$ from $\mathrm{Inv}-\chi^{2}\left(\hat{\nu },{\hat{\tau }}^{2}\right)$, where $\hat{\nu }=\nu +\sum_{k=1}^{K}n_{k}$ and ${\hat{\tau }}^{2}=\left({\nu \tau }^{2}+\sum_{k=1}^{K}\sum_{i=1}^{n_{k}}{\left(y_{\mathrm{ik}}-f\left(X_{\mathrm{ik}}\right)-Z_{\mathrm{ik}}^{⊤}\Lambda \Gamma b_{k}\right)}^{2}\right)/\left(\nu +\sum_{k=1}^{K}n_{k}\right)$.

#### 2.2.5 Posterior Computation and Inference

Posterior computation for the unified model (4) is conducted via a Gibbs sampler, employing the Bayesian backfitting algorithm described in Tan and Roy [23]. We define p(A|−) as the distribution of random variable A fully conditioning on all other variables, which is the target distribution for each step of the Gibbs sampler.

In Spike‐and‐Slab–based models, standard Gibbs sampling often performs poorly because of the strong coupling between $\lambda_{q}$ and the binary inclusion indicator $v_{q}$. The inclusion indicator divides the posterior distribution space of $\lambda_{q}$ into two disconnected regions, making it difficult for the sampler to move freely between them and frequently leading to poor mixing and convergence. To address this, we consider a partial collapse Gibbs sampler, which samples $v_{q}$ from the marginal conditional distribution by integrating $\lambda_{q}$ out, then samples other parameters from their fully conditional distributions. To address this, we adopt a partially collapsed Gibbs sampler: $v_{q}$ is sampled from its marginal conditional distribution by integrating out $\lambda_{q}$, followed by sampling all other parameters (including $\lambda_{q}$) from their uncollapsed full conditionals. To resolve incompatibility issues as discussed by van Dyk and Park [24], $\lambda_{q}$ is then sampled from its uncollapsed full conditional given $v_{q}$ and other parameters. It is crucial that these two steps are executed in this exact order without inserting any intermediate updates, as reordering or interleaving them would violate the validity of the sampler. This two‐step update can be viewed as a block Gibbs sampler: first drawing $v_{q}∼p\left(v_{q}\;|\;y_{\mathrm{ik}},f\left(X_{\mathrm{ik}}\right),Z_{\mathrm{ik}},\Gamma ,b_{k},\sigma^{2}\right)$, then drawing $\lambda_{q}∼p\left(\lambda_{q}|v_{q},-\right)$.

The full algorithm for the unified model (4) is provided in Algorithm 1. In steps 1 and 2, we update BART for fixed effects. Further details on BART updates are provided in previous studies [3, 23]. We update random effects related parts in steps 3–8. Steps 6–8 can be viewed as a whole step to update $v_{q}$ and $\lambda_{q}$. Since the posterior distribution of $v_{q}$ is complicated, to keep the notations simple in the step 6, we put the definitions of ${ℒ}_{1}$ and ${ℒ}_{0}$ are left in Supporting Information. Step 9 is used to update residual variance $\sigma^{2}$.

### 2.3 Two‐Step Approach

The unified mixed‐effects model provides a comprehensive framework for simultaneously selecting individual‐ and cluster‐level covariates. However, challenges arise when certain random predictors, denoted as $Z_{j}$ remain constant within clusters. The SODH data used in our case study (see Section 4) exhibit this characteristic. In such cases, near‐collinearity emerges between the random intercepts of the mixed‐effects model and the cluster‐level covariates $Z_{j}$. This issue stems from both components attempting to explain the same between‐cluster variation, which destabilizes the model and compromises the accurate selection of random predictors. As a result, the unified model may fail to effectively identify relevant cluster‐level covariates.

To address this limitation, we adopt a two‐step procedure that decouples random‐effects estimation from selection of cluster‐level covariates. First, we fit a BART model with a random‐intercept–only structure and extract the posterior means of the cluster intercepts as estimates of the latent cluster effects. Second, we regress these posterior means on the cluster‐level covariates $Z_{j}$ and apply a permutation‐calibrated selection procedure to assess covariate significance. By separating estimation from selection, this two‐stage approach mitigates collinearity, improves identification of relevant cluster‐level predictors, and yields more robust variable selection for random effects. Implementation details follow.

#### 2.3.1 Step 1: Fit BART With a Random‐Intercept–Only Specification

We apply a BART model to the response y using a random‐intercept–only version of the mixed‐effects model in Equation (4). The cluster‐specific random effect $\beta_{k}$ is a scalar intercept capturing between‐cluster heterogeneity. From this model, we extract the posterior mean vector $\hat{\beta }$ of the random intercepts β. Ideally, one would propagate the full posterior uncertainty in β (e.g., via credible intervals), but to control computational burden we carry forward the posterior mean $\hat{\beta }$ as a plug‐in estimate for subsequent analysis.

#### 2.3.2 Step 2: Permutation‐Based Selection for Cluster‐Level Covariates

We assess the importance of the cluster‐level covariates Z by modeling the estimated random intercepts as a function of Z. Specifically, treating $\hat{\beta }$ from Step 1 as the response, we fit a separate BART regression, $\hat{\beta }=\text{BART}\left(Z\right)+\epsilon$, to identify the subset of Z that best explains variation in $\hat{\beta }$. The three selection criteria used for fixed‐effects selection in Section 2.2.1 are reused here to select cluster‐level predictors. Statistical significance is evaluated via the permutation‐based procedure of Section 2.2.3: a predictor is deemed significant if its importance score exceeds the (1−α) quantile of the corresponding empirical null distribution.

Priors and posterior computation for the two‐step procedure parallel those of the unified model. In Step 1, we include only the random intercept and apply the same decomposition with a spike‐and‐slab prior to the associated random‐effect coefficients. In Step 2, the BART priors match those used for estimating fixed effects in the unified model, and categorical‐covariate handling follows the identical workflow.

We emphasize that the two‐step procedure is a general framework rather than a method tied to a specific modeling choice. While we have presented the two‐step approach using BART in both steps, the framework itself is modular. Any Bayesian (or frequentist) method capable of estimating cluster‐specific random intercepts can be employed in Step 1, and any variable selection method can be deployed in Step 2 to identify important cluster‐level covariates from the estimated intercepts. For example, one may substitute a Bayesian linear mixed model in Step 1 when the fixed‐effects relationship is believed to be linear, or employ a spike‐and‐slab Gibbs sampler in Step 2 instead of BART‐based permutation selection. This modularity allows researchers to tailor each step to the specific characteristics of their data, while preserving the core advantage of the two‐step design, where decoupling within‐cluster estimation from between‐cluster variable selection to mitigate near‐collinearity. In our simulation studies (Section 3), we demonstrate this flexibility by implementing both a BART‐based and a Bayesian linear two‐step variant, showing that the framework yields reliable cluster‐level selection under different Step 1 and Step 2 specifications.

## 3 Simulation

### 3.1 Simulation Design

We conducted comprehensive simulation studies to evaluate the performance of our proposed methods across a 2×2×2 factorial design, yielding eight distinct scenarios that vary along three dimensions: (i) *cluster structure* (balanced vs. unbalanced), (ii) *fixed‐effects specification* (nonlinear vs. linear), and (iii) *cluster‐level covariate structure* (within‐cluster varying vs. within‐cluster constant Z). This design enables systematic assessment of method performance under conditions that range from favorable (balanced clusters, nonlinear signals, within‐cluster varying Z) to challenging (unbalanced clusters, linear signals where BART's nonparametric flexibility confers no advantage, and cluster‐constant Z inducing near‐collinearity). For all eight scenarios, we applied PQL, Unified, Two‐step, and Sparse Dirichlet prior using the same tuning, priors, and implementation settings described in the main manuscript. For variable selection on the cluster‐level random‐effect covariates, we computed the same performance metrics as in the main text (Type I error, precision, recall, F1‐score, Type II error, and overall error rate), treating {1,2,3} as the set of true useful random‐effect covariates. More details will be shown in Section 3.2.

#### 3.1.1 Common Data‐Generating Components

Across all eight scenarios, the following components are shared. We simulated P=10 individual‐level covariates $X=\left(X_{1},\ldots ,X_{10}\right)$. Binary covariates $X_{1}$ and $X_{2}$ were independently generated from a Bernoulli(0.5) distribution, while the remaining continuous covariates $X_{3},\ldots ,X_{10}$ were independently drawn from standard normal distributions N(0,1).

The response variable y was simulated from the mixed‐effects model (1), with a random‐effects structure given by $Z_{\mathrm{ik}}\beta_{k}=\beta_{k0}+Z_{\mathrm{ik}1}\beta_{k1}+Z_{\mathrm{ik}2}\beta_{k2}+Z_{\mathrm{ik}3}\beta_{k3}$, where $\beta_{k0}$ is a random intercept and predictors $Z_{1}$, $Z_{2}$, and $Z_{3}$ represent the three informative random‐effects covariates. Additionally, three noise random‐effects covariates were independently generated from N(0,1), yielding a total of Q=7 candidate random predictors (including the intercept). The residual errors $\varepsilon_{\mathrm{ik}}$ were independently sampled from N(0,1). The overall variance ratio of random effects to total variance was set to approximately 10%–15%, a range that is realistic in many healthcare applications where cluster‐level heterogeneity contributes a meaningful but not dominant share of outcome variation. The random intercept was sampled independently from N(0,1), reflecting a nontrivial baseline level of between‐cluster heterogeneity that is not explained by observed cluster‐level covariates. This choice addresses reviewer concerns about the identifiability of cluster‐level effects and avoids artificially suppressing the random intercept contribution.

#### 3.1.2 Fixed‐Effects Specifications

We considered two fixed‐effects specifications to evaluate method performance under both complex and simple signal structures.

Under the *nonlinear* specification,

(6) $$f\left(X_{\mathrm{ik}}\right)=6X_{\mathrm{ik}1}+3X_{\mathrm{ik}5}-X_{\mathrm{ik}7}+X_{\mathrm{ik}7}^{2}+X_{\mathrm{ik}6}+X_{\mathrm{ik}6}X_{\mathrm{ik}8},$$

so that five individual‐level covariates $\left(X_{1},X_{5},X_{6},X_{7},X_{8}\right)$ are truly associated with the outcome and the remaining five $\left(X_{2},X_{3},X_{4},X_{9},X_{10}\right)$ are noise variables. This setting includes both a nonlinear main effect and an interaction term, thereby representing a more complex signal structure.

Under the *linear* specification,

(7) $$f\left(X_{\mathrm{ik}}\right)=6X_{\mathrm{ik}1}+3X_{\mathrm{ik}5}-X_{\mathrm{ik}7}+X_{\mathrm{ik}6},$$

four individual‐level covariates $\left(X_{1},X_{5},X_{6},X_{7}\right)$ are associated with the outcome, whereas $\left(X_{2},X_{3},X_{4},X_{8},X_{9},X_{10}\right)$ are noise variables. This setting corresponds to a correctly specified linear signal, providing a benchmark under which parametric linear mixed‐effects methods are not disadvantaged by model misspecification.

#### 3.1.3 Cluster Structure

We also considered two cluster designs. In the balanced design, there were K=50 clusters with $n_{k}=100$ individuals per cluster, yielding a total sample size of N=5,000. In the unbalanced design, there were K=35 clusters, with cluster sizes independently generated from Uniform{10,11,…,30}. Under the shared simulation seed, this produced a total sample size of N=648. This latter setting was chosen to resemble the structure of the case study data, which include 631 individuals distributed across clusters of unequal size, and to assess robustness under smaller and more heterogeneous cluster configurations.

#### 3.1.4 Structure of Random Predictors and Random Effects

We considered two cluster‐level covariate structures for the random predictors Z and their associated random coefficients β. In the first setting, the components of Z *varied* within clusters. Some random predictors associated with the individual‐level covariates X; specifically, we introduced moderate correlation (ρ=0.2) between $X_{5}$ and $Z_{1}$ and between $X_{6}$ and $Z_{2}$, while $Z_{3}$ was generated as an integer‐valued variable taking values in {0,…,3} by summing four independent Bernoulli(0.5) variables. All remaining components of Z were generated independently from N(0,1). The corresponding nonzero random coefficients were drawn independently across clusters from a multivariate normal distribution,

$$\beta_{k}∼ℳ{\mathrm{VN}}_{4}\left(0,{\tilde{\sum }}_{1}\right),$$

where ${\tilde{\sum }}_{1}$ was sampled from an inverse Wishart distribution with 6 degrees of freedom and identity scale matrix. Coefficients corresponding to unimportant predictors were set to zero. Predictor $Z_{3}$ was represented through dummy variables. In the second setting, the random predictors Z were *constant* within each cluster, creating a more challenging scenario because cluster‐level covariates may be nearly collinear with the random intercept. In this case, $Z_{1}$ and $Z_{2}$ were generated independently from N(0,1) at the cluster level, and $Z_{3}$ was generated as in the within‐cluster varying setting. To ensure sufficient between‐cluster variation in the random effects, we generated the random coefficients conditionally on $Z_{k}={\left(Z_{k1},Z_{k2},Z_{k3}\right)}^{T}$ according to

$$\beta_{k}|Z_{k}∼ℳ{\mathrm{VN}}_{3}\left(\left(\begin{array}{c} 2.5Z_{k1}\\ 2.5Z_{k2}\\ 2.0Z_{k3} \end{array}\right),{\tilde{\sum }}_{2}\right),$$

where

$${\tilde{\sum }}_{2}=\left(\begin{array}{ccc} 0.01 & -0.005 & -0.005\\ -0.005 & 0.01 & -0.005\\ -0.005 & -0.005 & 0.01 \end{array}\right).$$

This construction induces meaningful between‐cluster variation in the random effects despite the absence of within‐cluster variation in Z, thereby making variable selection feasible in the cluster‐constant setting. In addition, we also introduced moderate correlation (ρ=0.2) between $X_{5}$ and $Z_{1}$ and between $X_{6}$ and $Z_{2}$, while all remaining components of X were generated independently from N(0,1), except for $X_{1}$, which was binary. As above, $Z_{3}$ was dummy encoded.

#### 3.1.5 Summary of Simulation Scenarios

The simulation considered a 2×2×2 design defined by fixed‐effects specification (linear vs. nonlinear), cluster structure (balanced vs. unbalanced), and cluster‐level covariate structure (within‐cluster constant vs. within‐cluster varying). This yielded eight scenarios, each evaluated with three informative and three noise random‐effect covariates (Table 1).

**TABLE 1:** Performance metrics, summarized as mean (SD) over 250 simulation replicates, for the methods under eight simulation scenarios with three informative and three noise random‐effect covariates.

Scenario	Method	Fixed effects			Random effects			
		Recall	Precision	$F_{1}$	Recall	Precision	$F_{1}$	Type I error
Balanced design (K=50, $n_{k}=100$, N=5,000)								
Bal–NL–Var	Unified (MI)	0.90 (0.18)	0.99 (0.04)	0.94 (0.11)	1.00 (0.00)	1.00 (0.00)	1.00 (0.00)	0.00 (0.00)
	Two‐step	—			—			
	Sparse	0.83 (0.07)	1.00 (0.03)	0.90 (0.03)	1.00 (0.00)	1.00 (0.02)	1.00 (0.01)	0.00 (0.02)
	PQL	0.87 (0.09)	0.80 (0.15)	0.82 (0.09)	0.94 (0.17)	1.00 (0.03)	0.96 (0.12)	0.00 (0.00)
Bal–NL–Con	Unified (MI)	0.85 (0.22)	1.00 (0.02)	0.91 (0.14)	0.73 (0.24)	0.59 (0.19)	0.64 (0.19)	0.56 (0.31)
	Two‐step	0.85 (0.22)	1.00 (0.02)	0.91 (0.14)	0.85 (0.23)	0.96 (0.14)	0.88 (0.18)	0.04 (0.11)
	Sparse	0.82 (0.07)	0.99 (0.04)	0.90 (0.03)	0.70 (0.25)	0.69 (0.23)	0.66 (0.18)	0.40 (0.33)
	PQL	0.80 (0.01)	1.00 (0.01)	0.89 (0.01)	0.47 (0.22)	0.98 (0.08)	0.61 (0.17)	0.02 (0.11)
Bal–L–Var	Unified (MI)	1.00 (0.00)	0.99 (0.04)	1.00 (0.02)	1.00 (0.00)	1.00 (0.00)	1.00 (0.00)	0.00 (0.00)
	Two‐step	—			—			
	Sparse	0.94 (0.11)	0.97 (0.08)	0.95 (0.07)	1.00 (0.00)	1.00 (0.00)	1.00 (0.00)	0.00 (0.00)
	PQL	1.00 (0.00)	0.85 (0.15)	0.91 (0.09)	1.00 (0.00)	1.00 (0.00)	1.00 (0.00)	0.00 (0.00)
Bal–L–Con	Unified (MI)	1.00 (0.00)	1.00 (0.03)	1.00 (0.02)	0.76 (0.25)	0.59 (0.19)	0.64 (0.19)	0.57 (0.32)
	Two‐step	1.00 (0.00)	1.00 (0.03)	1.00 (0.02)	0.84 (0.22)	0.95 (0.14)	0.88 (0.19)	0.04 (0.11)
	Sparse	0.94 (0.11)	0.97 (0.07)	0.95 (0.07)	0.68 (0.25)	0.69 (0.23)	0.65 (0.19)	0.38 (0.33)
	PQL	1.00 (0.00)	1.00 (0.00)	1.00 (0.00)	0.46 (0.20)	0.98 (0.09)	0.60 (0.16)	0.02 (0.08)
Unbalanced design (K=35, $n_{k}∼\text{Uniform}\left\{10,\ldots ,30\right\}$, N=648)								
Unbal–NL–Var	Unified (MI)	0.84 (0.08)	0.99 (0.03)	0.91 (0.05)	0.78 (0.25)	1.00 (0.02)	0.85 (0.20)	0.00 (0.02)
	Two‐step	—			—			
	Sparse	1.00 (0.01)	0.98 (0.06)	0.99 (0.04)	0.79 (0.25)	1.00 (0.04)	0.87 (0.15)	0.01 (0.04)
	PQL	0.82 (0.10)	0.85 (0.18)	0.82 (0.10)	0.80 (0.27)	0.91 (0.17)	0.80 (0.19)	0.16 (0.31)
Unbal–NL–Con	Unified (MI)	0.80 (0.00)	1.00 (0.00)	0.89 (0.00)	0.33 (0.00)	0.99 (0.08)	0.50 (0.02)	0.00 (0.00)
	Two‐Step	0.80 (0.00)	1.00 (0.00)	0.89 (0.00)	0.68 (0.07)	0.98 (0.02)	0.81 (0.04)	0.01 (0.02)
	Sparse	1.00 (0.00)	0.98 (0.05)	0.99 (0.03)	0.56 (0.24)	0.78 (0.24)	0.61 (0.18)	0.24 (0.29)
	PQL	0.80 (0.00)	1.00 (0.00)	0.89 (0.00)	0.33 (0.00)	1.00 (0.00)	0.50 (0.00)	0.00 (0.00)
Unbal–L–Var	Unified (MI)	0.97 (0.08)	0.97 (0.07)	0.97 (0.06)	0.84 (0.23)	1.00 (0.02)	0.89 (0.18)	0.00 (0.02)
	Two‐step	—			—			
	Sparse	1.00 (0.00)	0.99 (0.05)	1.00 (0.03)	0.85 (0.22)	1.00 (0.02)	0.91 (0.14)	0.00 (0.03)
	PQL	1.00 (0.00)	0.64 (0.17)	0.77 (0.12)	0.93 (0.16)	0.93 (0.14)	0.92 (0.12)	0.10 (0.23)
Unbal–L–Con	Unified (MI)	0.97 (0.08)	0.99 (0.05)	0.98 (0.05)	0.68 (0.26)	0.70 (0.24)	0.67 (0.22)	0.34 (0.29)
	Two‐step	0.97 (0.08)	0.99 (0.05)	0.98 (0.05)	0.53 (0.31)	0.79 (0.37)	0.62 (0.32)	0.06 (0.13)
	TS (Bayes‐L)	0.98 (0.05)	1.00 (0.00)	0.99 (0.03)	0.72 (0.18)	0.83 (0.19)	0.77 (0.23)	0.12 (0.13)
	Sparse	1.00 (0.00)	0.98 (0.07)	0.99 (0.04)	0.56 (0.23)	0.79 (0.24)	0.62 (0.18)	0.23 (0.29)
	PQL	1.00 (0.00)	1.00 (0.01)	1.00 (0.01)	0.48 (0.22)	0.94 (0.16)	0.61 (0.16)	0.07 (0.19)

*Note*: The balanced and unbalanced designs differ substantially in total sample size (N=5,000 vs. N=648). Accordingly, comparisons across these two design settings reflect the combined effects of cluster imbalance and overall sample size; see Section 3. TS (Bayes‐L) denotes the two‐step approach using a Bayesian linear mixed‐effects model for selection. Results for VIP and VIP type are reported in Table S1.

Abbreviations: Bal, balanced; Con, within‐cluster constant; L, linear; NL, nonlinear; Unbal, unbalanced; Var, within‐cluster varying.

We generated 250 replicate datasets for each of the eight scenarios. Noted that The Two‐Step model is specifically designed to address the near‐collinearity that arises when cluster‐level covariates Z are constant within clusters. In the variant‐Z scenarios, within‐cluster variation in Z provides sufficient identifying information for the Unified model to perform joint selection directly, and the motivating collinearity problem does not arise. Accordingly, the Two‐Step model is not applied in the variant‐Z scenarios; results for these scenarios are reported for the Unified model, Sparse Dirichlet, and PQL only. In the constant‐Z scenarios, all four methods are compared. Additionally, we performed sensitivity analyses to assess the robustness of our methods to (i) increasing numbers of noise random predictors, (ii) variations in the permutation threshold α, (iii) larger numbers of individual‐level covariates, and (iv) different residual noise levels. See more details in 3.4.

### 3.2 Implementation Details and Evaluation Criteria

Although BART is frequently used for high‐dimensional fixed‐effect screening, the primary objective of our work is simultaneous selection of individual‐level fixed effects and cluster‐level random effects in hierarchical data, with rigorous error control via permutation calibration. Accordingly, our scalability experiments increase P to a moderate level (P=50) while preserving the mixed‐effects structure and the random‐effects selection task; the goal is to assess stability and runtime under additional nuisance covariates rather than to position the method as a specialized ultrahigh‐dimensional fixed‐effect selector.

To rigorously assess the effectiveness of our proposed methods, we compared them against the well‐established PQL approach [14]. This approach integrates penalized quasi‐likelihood estimation with adaptive lasso penalties. This method is specifically designed for the simultaneous selection of both fixed and random effects in generalized linear mixed models, making it a suitable benchmark for evaluating our proposed method. We implemented the PQL approach using the rpqlseq function from the rpql package in R, specifying adaptive lasso penalties for both fixed and random effects selection. A sequence of 100 tuning parameters ranging from $10^{-8}$ to 1 is provided as input to the function, to find the best penalty parameter. To provide a stronger linear mixed‐effects benchmark beyond PQL, we also considered glmmLasso, which performs $L_{1}$‐penalized estimation in generalized linear mixed models for fixed‐effect selection [25]. Although glmmLasso is widely used as a benchmark for sparse fixed‐effects recovery in GLMMs, it does not perform selection of the random‐effects structure. Instead, it assumes that the random‐effects structure is specified a priori and applies penalization only to the fixed‐effects coefficients. Accordingly, glmmLasso serves as an informative benchmark for fixed‐effects selection in linear mixed‐model settings, but it is not a direct comparator for joint multilevel selection of fixed and random effects.

The reviewer also raised mixed‐effects tree‐ and forest‐based methods for clustered data. We agree that these are important related approaches, including MERF (Mixed Effects Random Forest) [26] and RE‐EM Tree [27]. However, these methods were developed primarily for prediction in clustered settings rather than for multilevel variable selection with inferential summaries, such as posterior inclusion probabilities for fixed and random effects. Because our primary objective is simultaneous selection of fixed‐effect predictors and random‐effect components, including settings with cluster‐constant covariates, comparison with prediction‐oriented methods would not be aligned with the target estimands or evaluation criteria. We have therefore clarified this distinction in the revised manuscript and focused our simulation comparisons on methods that address the same selection objective.

For the hyperparameters of BART, we used the default settings. For the hyperparameters associated with the random effects in model (4), we specify $\gamma_{0}$ as a $\frac{Q\left(Q-1\right)}{2}$‐dimensional zero vector and define $R_{0}=0.5I$, where I is the identity matrix of size $\frac{Q\left(Q-1\right)}{2}\times \frac{Q\left(Q-1\right)}{2}$, similar as Chen's [15]. For each $\lambda_{q}$ (q=1,…,Q), Chen recommended to set $\theta_{q}=0$ and $s_{q}^{2}=30$, we further use Jeffrey's prior such that $c_{q}=0.5$, $d_{q}=0.5$ [15]. Although assigning different hyperparameters to each $\lambda_{q}$ would provide greater flexibility, we adopt the same values across all q=1,…,Q for simplicity. In particular, $c_{q}$ and $d_{q}$ imply a prior mean of 0.5 for each $\pi_{q}$, reflecting the assumption that, on average, each random effect is equally likely to be important or not. For each replicated set, we did $L_{\mathrm{rep}}=10$ repeats for original data and L=100 permutations with null data. α was set as 0.05. For each Bayesian model, we ran 10 000 iterations in total and kept the last 5000 iterations for variable selection. Because our workflow combines BART backfitting, a partially collapsed Gibbs sampler for spike‐and‐slab random effects, and a permutation procedure, we additionally assessed computational cost for all evaluated methods. For each method (Unified, Two‐step, Sparse Dirichlet, and PQL), we recorded the wall‐clock runtime per replicated dataset, including the total run time for the original data aggregated over $L_{\mathrm{rep}}=10$ chains and L=100 permuted datasets. All runtimes were measured in seconds using proc.time() in R and reported as mean with standard errors across the 250 replicates for each scenario. Experiments were run on a compute node with fixed hardware and software configuration to ensure a fair comparison across methods. We emphasize that permutation dominates runtime in the unified and two‐step permutation‐calibrated procedures, whereas the Sparse Dirichlet approach avoids permutations and therefore provides a substantially faster screening alternative when computational resources are limited.

To compare the methods, we evaluated their performance using standard classification metrics: Precision, Recall, $F_{1}$ score, and Type I error and are defined as below.

- $\text{Precision}=\frac{\mathrm{TP}}{\mathrm{TP}+\mathrm{FP}}$, where TP (true positives) is the number of correctly identified important variables, FP (false positives) is the number of noise variables incorrectly identified as important. The precision of a variable selection method refers to the proportion of selected variables that are truly important.
- $\text{Recall}=\frac{\mathrm{TP}}{\mathrm{TP}+\mathrm{FN}}$, where FN (false negatives) is the number of important variables that were missed. The recall of a variable selection method measures how well the method identifies all the truly important variables.
- $F_{1}=\frac{2\times \text{Precision}\times \text{Recall}}{\text{Precision}+\text{Recall}}$. The $F_{1}$ measure is the harmonic mean of precision and recall, summarizing the balance between selecting only important variables and not missing any.
- $\text{Type}\;I\;\text{error}=\frac{\mathrm{FP}}{\text{the number of noise predictors}}$. Type I error measures the proportion of irrelevant variables that are incorrectly selected by the variable selection method.

We computed these metrics separately for individual‐level covariates X and cluster‐level covariates Z, and reported their average values and standard deviation across 250 replicates to quantify each method's variable selection performance and uncertainty.

### 3.3 Simulation Results

**FIGURE 1:**
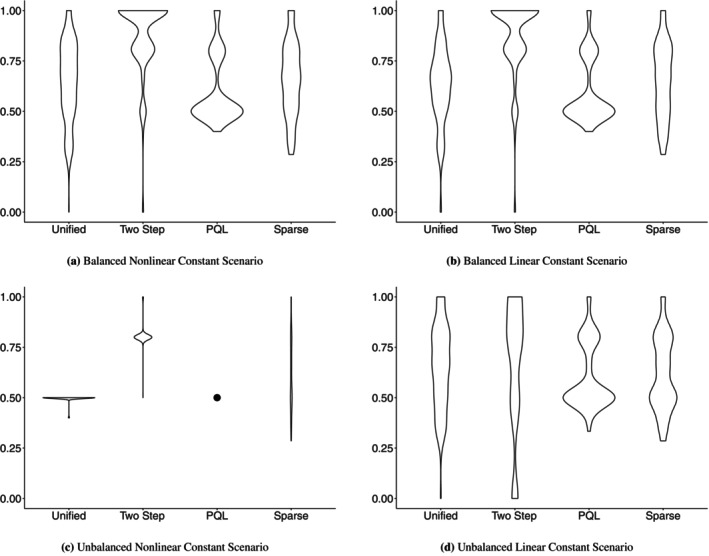
Violin plots of random‐effects selection $F_{1}$ scores for the four methods under the cluster‐constant scenarios. The top row corresponds to balanced cluster designs and the bottom row to unbalanced cluster designs. Within each row, panels are ordered from left to right as nonlinear‐constant and linear‐constant: (a) balanced nonlinear‐constant, (b) balanced linear‐constant, (c) unbalanced nonlinear‐constant, and (d) unbalanced linear‐constant. In panel (c), the PQL method yields an $F_{1}$ score of 0.50 with zero variance across replicates and therefore appears as a single point rather than a violin.

We applied Web Algorithm 1 in Supporting Information to each replicated dataset across all eight scenarios. Table 1 summarize Precision, Recall, $F_{1}$ score, and Type I error (with standard deviations) for the four methods—Unified (MI), Two‐Step (MI), PQL, and Sparse Dirichlet—across the balanced and unbalanced settings, respectively. For the constant settings, the results is also provided in Figure 1 for all comparison methods. Results based on VIP and within‐type VIP criteria are provided in the Table S1.

Across both balanced and unbalanced settings, the distinction between variant and constant Z was the single most influential factor for random‐effect selection. When Z varied within clusters, the Unified model consistently achieved near‐perfect random‐effect selection, with Recall, Precision, and $F_{1}$ scores all close to 1 and negligible Type I error. The Two‐Step model is not evaluated in the variant‐Z scenarios, as its two‐stage decomposition is motivated by the near‐collinearity between cluster‐constant covariates and random intercepts—a condition that does not arise when Z varies within clusters. Consequently, only the Unified model, Sparse Dirichlet, and PQL are compared in the variant‐Z settings.

In the Balanced–Nonlinear–Variant scenario, the Unified (MI) model achieved perfect random‐effect selection ($F_{1}=1.00$, Type I error =0.00). The Sparse Dirichlet method performed comparably ($F_{1}=1.00$, SD =0.01), while PQL achieved slightly lower random‐effect $F_{1}=0.96$ (SD =0.12), with Recall of 0.94 (SD =0.17) indicating that PQL occasionally missed a true random‐effect predictor. For fixed‐effect selection, the Unified model achieved $F_{1}=0.94$ (SD =0.11) with Precision of 0.99, the Sparse Dirichlet method achieved $F_{1}=0.90$ (SD =0.03) with slightly lower Recall (0.83), and PQL had the weakest performance with $F_{1}=0.82$ (SD =0.09), driven by notably lower Precision (0.80, SD =0.15). In the Balanced–Linear–Variant scenario, all three methods achieved perfect random‐effect selection ($F_{1}=1.00$, Type I error =0.00), confirming that when Z varies within clusters and the sample size is large, even PQL can reliably recover the random‐effects structure. For fixed effects, the Unified model achieved $F_{1}=1.00$ (SD =0.02), demonstrating that BART's regularizing priors prevent overfitting even when the true relationship is linear; the Sparse Dirichlet method achieved $F_{1}=0.95$ (SD =0.07); and PQL achieved $F_{1}=0.91$ (SD =0.09) with lower Precision (0.85, SD =0.15), indicating that PQL tended to over‐select noise covariates even under correct parametric specification.

When Z was constant within clusters, the Unified model's random‐effect selection deteriorated substantially. The near‐collinearity between cluster‐constant covariates and the random intercept destabilized the selection mechanism, leading to reduced Recall and elevated Type I error. In contrast, the Two‐Step model maintained strong performance in the balanced constant‐Z scenarios by decoupling within‐cluster and between‐cluster signals. For instance, in Balanced–Nonlinear–Constant setting, the Two‐Step (MI) method achieved random‐effect Recall of 0.85, Precision of 0.96, and $F_{1}=0.88$ (SD =0.18), with Type I error of 0.18. In contrast, the Unified model's random‐effect $F_{1}$ was only 0.64 (SD =0.19) in this scenario, with Precision of 0.59 (SD =0.19) and substantially elevated Type I error of 0.56 (SD =0.31), demonstrating that the near‐collinearity caused the Unified model to frequently select noise random predictors alongside the true ones. The Sparse Dirichlet method showed a similar pattern ($F_{1}=0.66$, SD =0.18, Type I error =0.40, SD =0.33). PQL was conservative, achieving high Precision (0.98, SD =0.08) but low Recall (0.47, SD =0.22), yielding $F_{1}=0.61$ (SD =0.17); it avoided false positives but frequently missed true random‐effect predictors. Under the linear specification (Blanced‐Linear‐Constant), performance was nearly identical, with random‐effect $F_{1}=0.88$ (SD =0.18) and Precision of 0.95, confirming that the Two‐Step approach is robust to the choice of fixed‐effects specification in the balanced setting. The Unified model again suffered from near‐collinearity in this setting, with random‐effect $F_{1}=0.64$ (SD =0.19), Precision of 0.59 (SD =0.19), and Type I error of 0.57 (SD =0.32)—nearly identical to its nonlinear counterpart, indicating that the near‐collinearity problem is driven by the constant‐Z structure rather than the fixed‐effects specification. The Sparse Dirichlet method ($F_{1}=0.65$, SD =0.19) and PQL ($F_{1}=0.60$, SD =0.16) again trailed the Two‐Step model. For fixed‐effect selection, although BART was also negatively affected, the Unified model and Sparse Dirichlet method remained competitive with PQL.

For fixed‐effect selection, the nonlinear specification (6) naturally advantaged the BART‐based methods (Unified, Two‐Step, and Sparse), which captured the interaction and quadratic terms without requiring explicit model specification. Under the linear specification (7), where parametric methods are correctly specified, PQL showed improved fixed‐effect selection relative to its nonlinear performance. Notably, the BART‐based methods remained competitive even in the linear setting, demonstrating that BART's regularizing priors prevent substantial overfitting when the true relationship is linear. The Sparse Dirichlet prior achieved relatively strong fixed‐effect selection across both specifications.

For random‐effect selection, the fixed‐effects specification had a more indirect influence. Comparing Balanced–Nonlinear–Constant and Balanced–Linear–Constant settings, the Two‐Step (MI) method achieved nearly identical random‐effect $F_{1}$ scores (0.88 in both cases), indicating that the cluster‐level selection in Step 2 is insensitive to whether Step 1 captures a nonlinear or linear fixed‐effects surface, provided the clusters are sufficiently large. However, the interaction between fixed‐effects complexity and cluster size became pronounced in the unbalanced setting, as discussed below.

Reducing cluster sizes from the balanced setting (K=50, $n_{k}=100$) to the unbalanced setting (K=35, $n_{k}\in \left\{10,\ldots ,30\right\}$) led to moderate performance decreases across all methods, as expected with the substantially smaller total sample size (N≈700 vs. N=5,000). Crucially, even under this more demanding design, the Two‐Step model continued to outperform the competing approaches. Before discussing the constant‐Z results, we note that the Unified model also maintained strong performance in the unbalanced variant‐Z scenarios. In Unbalanced–Nonlinear–Variant, although the Recall for random effects declines to 0.78 (SD =0.25) for the Unified model and 0.79 (SD =0.25) for the Sparse Dirichlet model, both methods still achieve competitive overall performance, with $F_{1}$ scores of 0.85 (SD =0.20) and 0.87 (SD =0.15), respectively. This drop in performance is driven by the severe imbalance in cluster sizes, which substantially reduces the effective within‐cluster information available to estimate random slopes; with fewer observations per cluster and greater heterogeneity across clusters, the model has much lower power to detect nonzero random‐slope variances, leading to more false negatives and thus lower Recall and overall performance. In comparison, PQL degraded most severely to $F_{1}=0.75$ (SD =0.19) with elevated Type I error of 0.16 (SD =0.31), indicating substantial instability consistent with PQL's known sensitivity to small, unbalanced clusters. For fixed‐effect selection, the Sparse Dirichlet method achieved the highest $F_{1}=0.99$ (SD =0.03), followed by the Unified model ($F_{1}=0.91$, SD =0.05) and PQL ($F_{1}=0.85$, SD =0.08). In Unbalanced–Linear–Variant, PQL was competitive with the BART‐based methods for random‐effect selection for the first time, achieving $F_{1}=0.92$ (SD =0.12), compared with the Unified model's $F_{1}=0.89$ (SD =0.18) and the Sparse Dirichlet's $F_{1}=0.91$ (SD =0.14). This is because PQL's parametric specification was correctly specified and within‐cluster variation in Z provided sufficient identifying information. However, PQL's fixed‐effect selection remained the weakest, with $F_{1}=0.77$ (SD =0.12) due to low Precision (0.64, SD =0.17), while the Unified model achieved $F_{1}=0.97$ (SD =0.06) and the Sparse Dirichlet achieved $F_{1}=1.00$ (SD =0.03). In Unbalanced–Nonlinear–Constant, the Two‐Step (MI) method achieved random‐effect Recall of 0.68, Precision of 1.00, and $F_{1}=0.81$ (SD =0.04). While this represents a decline from its balanced counterpart (Balanced‐Nonlinear‐Constant), ($F_{1}=0.88$), the perfect Precision in S6 is noteworthy: almost every random predictor selected by the Two‐Step model under the unbalanced nonlinear setting was a true signal. The reduced Recall reflects the inherent difficulty of estimating random intercepts from small clusters rather than a methodological limitation, as all competing methods exhibited comparable or greater degradation. Specifically, the Unified model suffered severely in this scenario, with random‐effect $F_{1}=0.50$ (SD =0.02) and Recall of only 0.33 (SD =0.00), consistently selecting only one of the three true random predictors. PQL showed a nearly identical pattern ($F_{1}=0.50$, Recall =0.33, Precision =1.00). The Sparse Dirichlet method achieved $F_{1}=0.61$ (SD =0.18) but with substantially higher Type I error (0.24, SD =0.29). These results are directly relevant to the case study, which features a comparable sample size and cluster structure, and suggest that the Two‐Step model remains a reliable tool even under realistic small‐sample conditions.

It is important to note that the balanced (N=5,000) and unbalanced (N=648) designs differ not only in cluster structure but also in total sample size by nearly an order of magnitude. Consequently, the performance differences observed between balanced and unbalanced scenarios reflect the combined effects of smaller clusters, heterogeneous cluster sizes, and a substantially reduced overall sample. This design choice was deliberate: rather than artificially equating total sample sizes, we specified the unbalanced setting to closely mirror the structure of our case study data. The fact that the Two‐Step model maintained meaningful random‐effect selection performance even under this realistic low‐N regime, which achieves $F_{1}=0.81$ in S6, for example, with perfect Precision, and it provides direct evidence that the method remains a reliable tool in the sample‐size ranges encountered in applied health research.

In Unbalanced–Linear–Constant scenario, the Two‐Step (MI) method achieved random‐effect Recall of 0.53 (SD =0.32), Precision of 0.79 (SD =0.37), and $F_{1}=0.62$ (SD =0.32). Although this represents a meaningful decline relative to the Two‐Step model's own performance in less demanding scenarios (Balanced‐Nonlinear‐Constant, $F_{1}=0.88$; Unbalanced‐Nonlinear‐Constant, $F_{1}=0.81$), it is important to note that all competing methods, including the Unified model, PQL, and the Sparse Dirichlet prior, experienced equal or greater degradation in this regime.

The reduced performance in Unbalanced‐Linear‐Constant can be attributed to a compounding of two data‐level difficulties that affect any method operating on this design. First, the unbalanced design with small clusters ($n_{k}$ as low as 10) and a total sample size of only N=648 provides substantially fewer observations from which to estimate cluster‐specific random intercepts, increasing the uncertainty in the Step 1 posterior means. Second, the linear fixed‐effects function is efficiently approximated by BART in Step 1, so the remaining between‐cluster variation that feeds into the estimated random intercepts is concentrated almost entirely in the intercept component itself. Together, these forces make the Step 2 response inherently noisy, amplifying identification difficulties for cluster‐level covariates regardless of the selection method employed. In contrast, under the nonlinear specification, BART's approximation of the more complex f(X) is less exact, leaving richer residual structure in the random intercepts that aids downstream cluster‐level selection. Additional experiments in which we increased the signal strength of the informative random‐effect covariates confirmed that $F_{1}$ can recover to approximately 0.80 in this scenario, indicating that the difficulty is fundamentally driven by the weak signal‐to‐noise ratio inherent in small, unbalanced clusters with a simple fixed‐effects surface, rather than by a methodological limitation of the two‐step framework itself. Importantly, the large standard deviations in Unbalanced‐Linear‐Constant also indicate that the Two‐Step model succeeds in many individual replicates with the average is pulled down by a subset of particularly difficult configurations, suggesting that in practice, the method can still provide useful guidance even in this challenging regime.

To further demonstrate the modularity of the two‐step framework in this challenging scenario, we implemented a fully Bayesian linear variant (Two‐Step (Bayesian‐Linear) in Table 1). In Step 1, a Bayesian linear mixed model was fitted with individual‐level covariates as fixed effects and a cluster‐specific random intercept to account for within‐cluster correlation. The model was implemented using the brm function from the brms package in R [28, 29], which provides a flexible interface for specifying Bayesian multilevel models with default weakly informative priors on fixed‐effect coefficients and variance components. The Bayesian framework was advantageous because it allowed coherent uncertainty quantification for both fixed and random effects through the full posterior distribution, producing well‐calibrated posterior mean random intercepts even in the small‐sample unbalanced setting. In Step 2, these posterior mean random intercepts were modeled as a function of the cluster‐level covariates using a Bayesian spike‐and‐slab variable selection procedure [30, 31, 32]. The spike‐and‐slab prior is a classical Bayesian approach to variable selection that assigns each regression coefficient a mixture prior consisting of a point mass at zero (the “spike”) and a diffuse continuous distribution (the “slab”). Formally, for each cluster‐level coefficient $\phi_{j}$, the prior takes the form $\phi_{j}|\omega_{j}∼\left(1-\omega_{j}\right)\;\delta_{0}+\omega_{j}\;N\left(0,\tau^{2}\right)$, where $\omega_{j}∼\text{Bernoulli}\left(\pi_{0}\right)$ is a binary inclusion indicator, $\delta_{0}$ denotes the Dirac mass at zero, and $\tau^{2}$ controls the prior variance of nonzero coefficients. A Gibbs sampler alternates between updating the inclusion indicators $\omega_{j}$ from their conditional Bernoulli distributions and updating the nonzero coefficients from their conditional normal distributions [31, 32]. This formulation enables direct variable selection by assigning positive posterior probability to exact exclusion ($\phi_{j}=0$), thereby distinguishing negligible effects from substantively important ones, which is a property that is particularly useful when the number of candidate cluster‐level covariates is small and some may be entirely irrelevant. A covariate is selected if its posterior inclusion probability $\mathrm{Pr}\left(\omega_{j}=1|\text{data}\right)$ exceeds 0.5, corresponding to the median probability model [32]. This Bayesian linear two‐step variant achieved random‐effect Recall of 0.72 (SD =0.18), Precision of 0.83 (SD =0.19), and $F_{1}=0.77$ (SD =0.23), with Type I error of 0.12 (SD =0.13)—a substantial improvement over the BART‐based Two‐Step (MI) variant ($F_{1}=0.62$) and all other competing methods in this scenario. The improvement arises because, when the true fixed‐effects surface is linear, a correctly specified linear mixed model in Step 1 estimates the random intercepts more efficiently than BART, yielding a less noisy response for Step 2 and thereby improving downstream cluster‐level selection. This result highlights that the two‐step framework is genuinely modular, and researchers should match the Step 1 model to the expected complexity of the fixed‐effects relationship.

Across all eight scenarios, the following patterns emerged, leading to clear practical guidance for applied researchers.

When Z varies within clusters, the Unified model is the recommended approach. It achieved the best joint fixed‐ and random‐effect selection by leveraging within‐cluster variation to distinguish informative random predictors, with $F_{1}\geq 0.85$ for random‐effect selection in every variant‐Z scenario and perfect selection ($F_{1}=1.00$) in the balanced settings. The Sparse Dirichlet method provides a competitive and computationally cheaper alternative in the unbalanced variant settings. PQL was reliable for random‐effect selection only when the fixed‐effects specification was linear and Z varied within clusters; under nonlinear fixed effects, its random‐effect selection degraded substantially, particularly in the unbalanced design ($F_{1}=0.75$, Type I error =0.16). The Two‐Step model is unnecessary in this setting, as the motivating near‐collinearity problem does not arise.

When Z is constant within clusters, the Two‐Step model is the recommended approach. It consistently outperformed all competing methods for random‐effect selection, achieving $F_{1}$ scores of 0.88 in both balanced constant‐Z scenarios and $F_{1}=0.81$ in the unbalanced nonlinear constant setting. The Two‐Step model's key advantage is its ability to decouple within‐cluster and between‐cluster signal, thereby avoiding the near‐collinearity that hampered both the Unified model and the Sparse Dirichlet method (both of which exhibited Type I errors exceeding 0.30 in most constant‐Z scenarios). Even in the most demanding configuration (Unbal–L–Con), the Two‐Step framework, when paired with an appropriately specified Step 1 and Step 2 models, achieved $F_{1}=0.77$, which the highest among all methods. Moreover, the framework's modularity allows researchers to match the Step 1 model to the expected complexity of the fixed‐effects relationship, providing additional flexibility in challenging scenarios.

For rapid screening or computationally constrained settings, the Sparse Dirichlet prior offers a practical alternative. It achieved competitive fixed‐effect selection with substantially lower computational cost (a single MCMC chain with no permutations) and can serve as an initial exploratory tool before applying the more rigorous permutation‐based approaches. However, its random‐effect selection was consistently weaker than the Two‐Step model, particularly in constant‐Z settings, so it should not be relied upon as a final selection method when cluster‐level identification is a primary objective.

PQL consistently underperformed the BART‐based methods for both fixed‐ and random‐effect selection, especially under nonlinear fixed effects where its parametric assumptions were violated. While PQL remains appropriate as a computationally efficient baseline for correctly specified linear models with variant Z, it is not recommended when the fixed‐effects structure may be nonlinear or when cluster‐level covariates are constant within clusters.

In summary, the proposed methods provide a flexible and complementary toolkit: the Unified model for variant‐Z settings, the Two‐Step model for constant‐Z settings, and the Sparse Dirichlet prior for rapid preliminary screening. Together, they consistently outperform existing alternatives across a wide range of realistic data configurations.

For computational efficiency, the mean wall‐clock runtime per replicate (along with the interquartile range) is measured for each method using proc.time() in R. All computations were performed on the cores of the Intel Xeon Gold 6538Y+ processor (2.20 GHz) with 3 GB of memory. Runtimes include all stages of the analysis: for the Unified model, the $L_{\mathrm{rep}}=10$ chains on the original data plus L=100 permutation fits; for the Two‐Step model, the Step 1 fit, the $L_{\mathrm{rep}}$ repetitions, and the L permutation fits in Step 2; for PQL, the sequential tuning‐parameter search; and for the Sparse Dirichlet method, the single MCMC run. The Unified method required the longest computation time. Both the sample size and the number of random‐effect variables substantially influence the overall time cost. On average, it took approximately 10 h in balanced scenarios and about 3 h in unbalanced scenarios when there were 6 random‐effect variables. When the number of random‐effect variables increased to 9, the computation time rose to roughly 15 h. The Two‐Step (MI) method took longer to run under balanced designs than unbalanced ones. Specifically, in the balanced scenarios, it averaged about 5.3 min for the Balanced‐Nonlinear‐Constant case and 5.2 min for the Balanced‐Linear‐Constant. In the unbalanced scenarios, runtimes were roughly halved, coming in at 2.3 min for Unbalanced‐Nonlinear‐Constant and 2.4 min for Balanced‐Linear‐Constant. Compared to the unified model, the sparse model was much more quicker, only taking about 10.4 min in the balanced scenario and 7 min in the unbalanced scenario, because it requires only a single MCMC chain without permutations, making it attractive for rapid screening. PQL was also computationally efficient due to its optimization‐based fitting, taking less than 1 min in every scenario.

### 3.4 Sensitivity Analyses

#### 3.4.1 Varying the Number of Noise Random Predictors

To assess how increasing the ratio of noise to informative random predictors affects random‐effect selection performance, we varied the number of noise covariates in Z from 3 to 6 under the Balanced–Nonlinear–Constant setting. Table 2 reports the performance metrics for random‐effect selection from the Two‐Step model. Across all noise levels, the Two‐Step (MI) method maintained stable random‐effect $F_{1}$ scores: 0.88 with 3 noise covariates, rising slightly to 0.90 with 4 noise, and stabilizing at 0.89 with 5 and 6 noise covariates. Precision showed a modest trade‐off, declining from 0.96 (3 noise) to 0.87 (6 noise) as more candidate covariates competed to explain between‐cluster variation, but Recall simultaneously improved from 0.85 to 0.93, yielding overall stable $F_{1}$ scores. Type I error remained controlled at 0.16–0.18 across all noise levels. These results demonstrate the robustness of the Two‐Step approach to increasing dimensionality in the random‐effects space. The full result table is provided in Table S2. Figure S1 further demonstrates that MI's performance remained stable across increasing noise levels when selecting random effects.

**TABLE 2:** Performance metrics for varying numbers of noise random predictors under Balanced–Nonlinear–Constant. All settings use 3 useful Z.

Noise Z	Method	Fixed effect			Random effect			
		Recall (SD)	Precision (SD)	$F_{1}$ (SD)	Recall (SD)	Precision (SD)	$F_{1}$ (SD)	Type I error (SD)
3 Noise	Unified (MI)	0.85 (0.22)	1.00 (0.02)	0.91 (0.14)	0.76 (0.25)	0.60 (0.20)	0.65 (0.20)	0.56 (0.32)
	Two‐step (MI)	0.85 (0.22)	1.00 (0.02)	0.91 (0.14)	0.85 (0.23)	0.96 (0.14)	0.88 (0.18)	0.04 (0.11)
	Sparse	0.82 (0.07)	0.99 (0.04)	0.90 (0.03)	0.70 (0.25)	0.69 (0.23)	0.66 (0.18)	0.40 (0.33)
	PQL	0.80 (0.01)	1.00 (0.01)	0.89 (0.01)	0.47 (0.22)	0.98 (0.08)	0.61 (0.17)	0.02 (0.11)
4 Noise	Unified (MI)	0.86 (0.22)	1.00 (0.02)	0.91 (0.14)	0.69 (0.24)	0.55 (0.21)	0.59 (0.19)	0.49 (0.29)
	Two‐step (MI)	0.86 (0.22)	1.00 (0.02)	0.91 (0.14)	0.90 (0.19)	0.93 (0.13)	0.90 (0.14)	0.06 (0.11)
	Sparse	0.83 (0.07)	0.99 (0.03)	0.90 (0.03)	0.67 (0.24)	0.49 (0.15)	0.56 (0.16)	0.31 (0.25)
	PQL	0.80 (0.00)	1.00 (0.01)	0.89 (0.01)	0.48 (0.21)	0.97 (0.11)	0.61 (0.17)	0.02 (0.08)
5 Noise	Unified (MI)	0.86 (0.22)	1.00 (0.02)	0.91 (0.14)	0.69 (0.24)	0.53 (0.19)	0.58 (0.18)	0.43 (0.25)
	Two‐step (MI)	0.86 (0.22)	1.00 (0.02)	0.91 (0.14)	0.91 (0.19)	0.90 (0.16)	0.89 (0.16)	0.07 (0.11)
	Sparse	0.83 (0.07)	1.00 (0.03)	0.90 (0.03)	0.67 (0.26)	0.47 (0.15)	0.54 (0.17)	0.28 (0.22)
	PQL	0.80 (0.00)	1.00 (0.00)	0.89 (0.00)	0.47 (0.20)	0.95 (0.14)	0.60 (0.15)	0.03 (0.10)
6 Noise	Unified (MI)	0.92 (0.17)	1.00 (0.02)	0.95 (0.12)	0.65 (0.25)	0.50 (0.20)	0.54 (0.19)	0.38 (0.23)
	Two‐step (MI)	0.92 (0.17)	1.00 (0.02)	0.95 (0.12)	0.93 (0.17)	0.87 (0.16)	0.89 (0.15)	0.08 (0.10)
	Sparse	0.83 (0.08)	0.99 (0.03)	0.91 (0.04)	0.69 (0.25)	0.44 (0.14)	0.52 (0.16)	0.32 (0.23)
	PQL	0.80 (0.00)	1.00 (0.00)	0.89 (0.00)	0.49 (0.21)	0.96 (0.13)	0.61 (0.16)	0.03 (0.08)

#### 3.4.2 Sensitivity to the Permutation Threshold

We conducted a sensitivity analysis for different permutation thresholds α∈{0.005,0.01,0.05,0.1} under the Balanced–Nonlinear–Constant setting using the Two‐Step model with MI. Figure S3 shows how $F_{1}$ score, Precision, Recall, and Type I error for random‐effect selection changed across these thresholds.

As α increased from 0.005 to 0.1, random‐effect $F_{1}$ scores improved steadily from 0.82 (SD =0.26) to 0.90 (SD =0.15). This improvement was driven primarily by gains in Recall, which increased from 0.78 to 0.88, while Precision remained relatively stable (0.92 to 0.95). Type I error declined from 0.23 to 0.15 as the more permissive threshold allowed more true positives. The narrowing standard deviations with increasing α (from 0.26 at α=0.005 to 0.15 at α=0.1) also indicate that the selection procedure becomes more stable with a less stringent threshold. Setting α=0.05 yielded $F_{1}=0.88$ with a good balance between Precision (0.96) and Recall (0.85), and we adopted this as the default threshold throughout. The full table is provided in Table S3 and Figure S3 visualizes the results as well.

Across all metrics except Precision, performance generally improved with increasing α (less stringent threshold), though the magnitude and direction of changes differed by metric and noise level. $F_{1}$ scores increased steadily for all settings as α increased, with slower improvements in high‐noise settings. Recall and Type I errors improved monotonically with α, as increasing α allowed more true and false positives to be captured. Precision showed a more nuanced trend: while it improved with α in lower‐noise scenarios, it declined modestly in higher‐noise settings, reflecting the trade‐off between capturing important covariates and including irrelevant ones. To balance Precision and Recall, we set α=0.05 as the default threshold throughout, ensuring that neither Precision nor Recall substantially declines in most cases.

#### 3.4.3 Scalability and Noise Robustness

To evaluate scalability and robustness, we conducted two additional experiments under the Balanced–Nonlinear–Constant setting, varying (i) the number of individual‐level covariates P∈{10,50} and (ii) the residual noise standard deviation $\sigma_{\varepsilon }\in \left\{0.1,0.5,1.0\right\}$, while maintaining the same multilevel structure, three informative random predictors, and the fixed significance threshold α=0.05.

When P was increased from 10 to 50 (with 45 additional noise individual‐level covariates drawn independently from N(0,1)), random‐effect selection for the Two‐Step (MI) method remained remarkably stable. At $\sigma_{\varepsilon }=1.0$, the Two‐Step (MI) method achieved random‐effect $F_{1}=0.88$ (SD =0.18) with P=10 and $F_{1}=0.87$ (SD =0.20) with P=50—a negligible difference. The same pattern held across all noise levels: at $\sigma_{\varepsilon }=0.1$, $F_{1}$ was 0.88 vs. 0.87; at $\sigma_{\varepsilon }=0.5$, $F_{1}$ was 0.89 vs. 0.88. This stability is a direct consequence of the Two‐Step architecture: because the cluster‐level selection in Step 2 operates on the reduced‐dimension space of estimated random intercepts (one per cluster) rather than the full P‐dimensional covariate set, the increase in individual‐level dimensionality does not propagate into the random‐effect selection stage. Precision remained high (0.94–0.95) and Recall stable (0.84–0.85) regardless of P.

Varying $\sigma_{\varepsilon }$ from 0.1 to 1.0 had essentially no effect on random‐effect selection performance. For P=10, the Two‐Step (MI) method achieved $F_{1}=0.88$ at $\sigma_{\varepsilon }=0.1$, $F_{1}=0.89$ at $\sigma_{\varepsilon }=0.5$, and $F_{1}=0.88$ at $\sigma_{\varepsilon }=1.0$. For P=50, the corresponding $F_{1}$ scores were 0.87, 0.88, and 0.87. Type I error also remained consistently controlled between 0.17 and 0.19 across all configurations. This insensitivity to residual noise arises because, in the balanced design with large clusters ($n_{k}=100$), the posterior means in Step 1 are estimated with sufficient precision regardless of the individual‐level noise level. The between‐cluster signal attributable to Z is thus preserved through Step 2 even at higher noise levels. Table S4 shows the full results and Figure S2 also demonstrates the result in the plots.

Overall, these results demonstrate that the Two‐Step model's random‐effect selection is highly robust to both increasing individual‐level dimensionality and varying residual noise, provided the clusters are sufficiently large. The practical implication is that researchers can apply the Two‐Step approach with confidence in moderate‐ to high‐dimensional settings without concern that enlarging the predictor set will compromise cluster‐level variable identification.

## 4 Case Study

We applied our methods to data from the 2020 New Jersey Social Determinants of Health (NJ SDOH) database linked with the Pathways to Healthy Aging in African Americans (Pathways) study [33]. The NJ SDOH database assembles cluster‐level indicators from the U.S. Census Bureau, the American Community Survey (ACS), and the Centers for Medicare & Medicaid Services (CMS), spanning social, economic, educational, health‐care, and geographic domains (e.g., ZIP‐code level median household income, median rent, percentage of households receiving food assistance, and poverty rates). The Pathways study examines cognitive health and its associations with lifestyle, behavioral, biological, and sociodemographic factors among African Americans aged 60 years and older, with key individual‐level measures including sleep quality, body mass index, aerobic fitness, and cognitive outcomes. Our primary outcome was generalization accuracy on the Concurrent Discrimination and Transfer Task, a cognitive marker sensitive to preclinical Alzheimer's disease related changes in hippocampal and medial temporal circuits. The linked dataset comprised 631 individuals from 83 ZIP codes in New Jersey, predominantly in Essex County, enabling joint evaluation of individual‐ and cluster‐level determinants of cognitive health in older African American adults.

We sought to identify individual‐level predictors of generalization accuracy and to quantify the contribution of cluster‐level social determinants. The analytic sample comprised 400 of 564 participants with non‐missing generalization‐accuracy scores. Guided by prior work linking Alzheimer's disease to education, access to health care, and the built environment [34], we prespecified 19 candidate predictors: nine individual‐level variables (seven continuous, two categorical) and ten cluster‐level variables (all continuous); definitions appear in Supporting Information: Appendix G. Missingness among individual‐level covariates was substantial (1.00%–43.44%). Participants were distributed across 79 ZIP codes, 38 of which contained a single observation, yielding sparse clusters. To mitigate sparsity, we excluded ZIP codes with only one observations or have no cluster‐level social determinants (retaining 35 ZIP codes) and also aggregated ZIP codes into 24 city‐level regions. These steps yielded two analytic datasets (total n=523) that differed only in their cluster definition: one at the ZIP‐code level (where all cluster‐level variables are constant within clusters) and one at the city level (where cluster‐level variables may vary within clusters due to aggregation from finer geographic units). Even at the city level, only East Orange, Newark, Plainfield and Jersey City encompass multiple ZIP codes, so residual near‐collinearity may remain.

We applied the PQL approach and our proposed unified model and two‐step model to perform variable selection on both individual‐level and cluster‐level variables, using the same hyperparameter settings as in the simulation studies. To address the missing data in the dataset, we performed 100 imputations using a two‐level joint modeling multiple imputation procedure, assuming a joint multivariate normal distribution for the partially observed data. The imputation was implemented using the jomo.smc function from the jomo package in R [35], and variable selection methods were applied to the average imputed dataset, in order to reducing the instability of the final result.

In addition to evaluating variable selection results, we assessed the predictive performance of each method's selected submodel, using approximate leave‐one‐out cross‐validation (LOO) and the expected log pointwise predictive density (ELPD) [36]. The ELPD is the expected log predictive density for new data, that $\text{ELPD}=\sum_{i=1}^{n}\log\int p\left(y_{i}|\theta \right)\;p\left(\theta |y\right)\;d\theta$ for data $y_{1},\ldots ,y_{n}$ with parameters θ. The Bayesian LOO estimate of out‐of‐sample predictive fit is ${\text{elpd}}_{\mathrm{loo}}=\sum_{i=1}^{n}\log p\left(y_{i}|y_{-i}\right)$, where $p\left(y_{i}|y_{-i}\right)=\int p\left(y_{i}|\theta \right)p\left(\theta |y_{-i}\right)\;d\theta$ is the leave‐one‐out predictive density given the data without the ith data point. Higher value of ${\text{elpd}}_{\mathrm{loo}}$ indicates better predictive performance and it can be efficiently estimated through Pareto smoothed importance sampling (PSIS), using a generalized Pareto distribution fit to the upper tail of the distribution of the simulated importance ratios to stabilize importance weights [37]. For each set of selected variables from the three models, we refitted Bayesian linear mixed‐effects models on the average dataset. Model fitting was performed using the brm function from the brms package in R. ${\text{elpd}}_{\mathrm{loo}}$ calculation is through loo function in the same package. For benchmarking purposes, we also evaluated a full model including all variables to compare the predictive performance of the variable selection methods.

Table 3 summarizes variable‐selection results for the unified model, the two‐step model, and the PQL benchmark under two clustering structures (ZIP‐code level and city level). In the ZIP‐code level dataset, both the unified and two‐step models consistently selected the same individual‐level variables: body mass index (BMI) and estimated aerobic fitness (VO_2_max). The Sparse Dirichlet method tended to select more individual‐level variables–gender, years of education (edu), self‐reported sleep quality (psqi_sleepquality) and estimated aerobic fitness (VO_2_max)–while the PQL method only selected estimated aerobic fitness (VO_2_max). At the cluster level, only the two‐step model selected the percentage of the population aged 25 and over with a high‐school diploma (ACS_PCT_HS_GRADUATE) while other models selected nothing. In the city‐level dataset, the unified model and the two‐step model continued selecting the same variables: body mass index (BMI), years of education (edu) and estimated aerobic fitness (VO_2_max); the Sparse Dirichlet method still tended to select more individual‐level variables: gender, years of education (edu), self‐reported sleep quality (psqi_sleepquality) and estimated aerobic fitness (VO_2_max); the PQL model only selected estimated aerobic fitness (VO_2_max). Cluster‐level selection diverged more at this level: the unified models selected the percentage of the population aged 25 and over with a high‐school diploma (ACS_PCT_HS_GRADUATE), while the Sparse Dirichlet model in addition selected Median year structure built of housing units (ACS_MEDIAN_YEAR_BUILT). The two‐step model selected median household income (ACS_MEDIAN_HH_INC) and the percentage of population with a disability (ACS_PCT_DISABLE). As in the ZIP‐code level analysis, the PQL model did not select cluster‐level variables.

**TABLE 3:** Variable selection list for unified, two‐step, Sparse Dirichlet and PQL models in real data application.

Dataset	Model	Individual‐level variable selection	Cluster‐level variable selection
Zip code level (constant cluster‐level)	Unified model	BMI, VO_2_max	None
	Two‐step model	BMI, VO_2_max	ACS_PCT_HS_GRADUATE
	Sparse Dirichlet	Gender, edu, psqi_sleepquality, VO_2_max	None
	PQL	VO_2_max	None
City level (variant cluster‐level)	Unified model	BMI, edu, VO_2_max	ACS_PCT_HS_GRADUATE
	Two‐step model	BMI, edu, VO_2_max	ACS_MEDIAN_HH_INC, ACS_PCT_DISABLE
	Sparse Dirichlet	Gender, edu, psqi_sleepquality, VO_2_max	ACS_MEDIAN_YEAR_BUILT, ACS_PCT_HS_GRADUATE
	PQL	VO_2_max	None

Predictive performance, evaluated using elpd_loo, is reported for both datasets. In the ZIP‐code level dataset, the two‐step model achieved an elpd_loo of −729.1 (SD = 15.4), the unified model −732.8 (SD = 15.4), and the Sparse Dirichlet model −727.8 (SD = 15.8); the PQL model was lower at −734.8 (SD = 15.0). In the city‐level dataset, the unified and Sparse Dirichlet models were comparable (−723.7 [SD = 15.7] vs. –724.4 [SD = 15.8]), the PQL model was −734.8 (SD = 15.0), and the two‐step model was −727.7 (SD = 15.7). Pairwise elpd_loo differences among models are provided in Table 4. Overall, our BART‐based approaches generally outperformed PQL, although differences were small and not statistically significant, underscoring the value of modeling cluster‐level structure even when selected covariates are sparse.

**TABLE 4:** Pairwise ${\text{elpd}}_{\mathrm{loo}}$ difference (standard deviation) between different models in zip code level and city level datasets. Each cell shows the difference calculated as the model in the left column minus the model in the top row.

	Zip code level			City level		
	Unified	Two‐step	Sparse Dirichlet	Unified	Two‐step	Sparse Dirichlet
Two‐step	3.8 (4.4)	—	—	−4.0 (3.2)	—	—
Sparse Dirichlet	5.0 (6.9)	1.3 (8.0)	—	−0.7 (5.7)	−3.3 (6.6)	—
PQL	−2.0 (3.2)	−5.8 (5.4)	−7.0 (6.1)	−11.1 (19.5)	−7.2 (19.5)	−10.5 (19.8)

**FIGURE 2:**
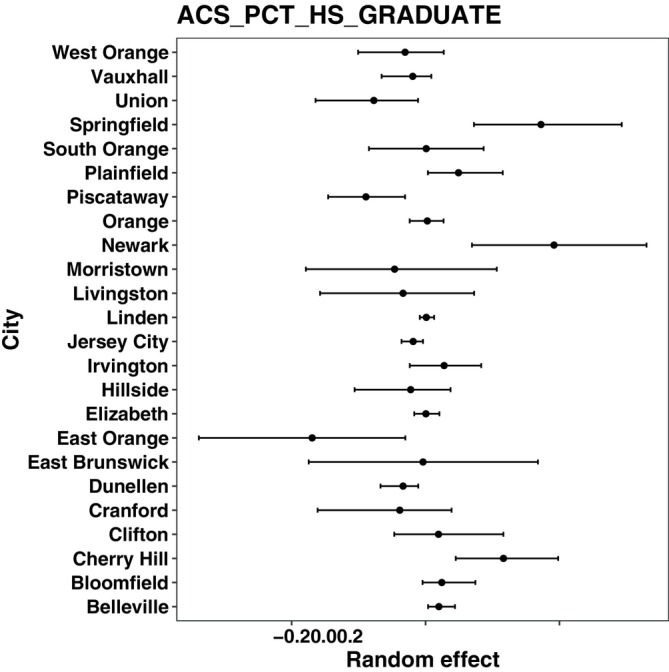
Estimated random effects for ACS_PCT_HS_GRADUATE, based on imputed city level datasets.

We emphasize that the primary goal of this work is multilevel variable selection (identifying important individual‐ and cluster‐level predictors and random‐effect components with uncertainty quantification), rather than maximizing out‐of‐sample predictive accuracy. In this real‐data application, several features constrain the magnitude of achievable predictive differences across methods. First, the effective sample size for learning cluster‐level effects is small: after filtering, the ZIP‐code analysis contains only 35 clusters and the city‐level analysis contains 24 clusters, with many clusters still sparse. Second, cluster‐level covariates exhibit limited variation (and are constant within clusters at the ZIP‐code level), so the incremental predictive contribution of cluster‐level terms beyond a random intercept is necessarily modest. Third, measurement noise in the cognitive outcome and substantial missingness (1.00%–43.44% in individual‐level covariates) further attenuate signal, so even materially different selection rules can yield similar predictive scores. Under these constraints, ${\text{elpd}}_{\mathrm{loo}}$ is not expected to separate methods strongly. Despite modest predictive differences, the case study demonstrates practical advantages aligned with our methodological aims. First, our framework yields interpretable selections at both levels (individual and cluster) while explicitly accounting for within‐cluster correlation and between‐cluster heterogeneity; in contrast, competing approaches tended to select no cluster‐level variables (Table 3). Second, the method is robust to near‐collinearity and sparse clustering. The unified model and, in particular, the two‐step procedure remain operational and yield stable cluster‐level selections even when cluster‐level predictors are constant within clusters and many clusters are small. Third, the estimated random effects and their variation across cities (Figure 2) provide a transparent depiction of between‐cluster heterogeneity, which is a central scientific target in multi‐site public‐health studies.

We further examined associations between the outcome (chooseprobeaccavg) and the individual‐level predictors selected by our methods. Supporting Information: Appendix F reports estimated fixed effects at the ZIP‐code and city levels and they are quite consistent. Though not significant, BMI was positively associated with cognitive performance. Prior literature suggests that in older adults, higher BMI may be weakly associated with better cognitive performance, possibly reflecting better nutritional status or the absence of disease‐related weight loss [38, 39, 40]. Both years of education (edu) and estimated aerobic fitness (VO_2_max) are significantly positively associated with cognitive performance. Studies show an association between a higher level of education and better brain health [34]. The current research also shows that higher VO_2_max is associated with better executive function performance [41]. To assess the face validity of cluster‐level selections, we plotted the city‐specific random effects for ACS_PCT_HS_GRADUATE (Figure 2). The effects varied substantially across cities, with the largest departures in Newark and East Orange–locales comprising multiple ZIP codes–highlighting the importance of random effects to capture between‐city heterogeneity.

In summary, we combined individual‐level data from the Pathways study with cluster‐level information from the New Jersey SDOH database to identify predictors of cognitive performance. Across both ZIP‐code and city‐level datasets, BMI and VO_2_max were the most frequently selected individual‐level predictor; at the city level, edu was additionally included. For cluster‐level effects, the two‐step model identified multiple socioeconomic variables in the city‐level analysis, whereas the unified model selected the proportion of adults aged 25+ with only a high‐school diploma. The PQL approach was conservative at both the individual and cluster‐level selection. Predictive performance (elpd_loo) was comparable for the two‐step and unified models, with PQL consistently underperforming. Collectively, these results underscore the value of flexible Bayesian methods for joint selection of individual‐ and cluster‐level predictors in cognitive health research.

## 5 Discussion

In recent healthcare research, repeated measurements and multi‐location data necessitate advanced variable selection methods in mixed‐effects models. While tree‐based approaches like BART have proven effective for fixed effects, selecting random effects remains challenging, especially when constant cluster‐level covariates coexist with random intercepts, leading to near‐collinearity issues. To address this, we proposed a fully Bayesian unified framework for simultaneous selection that integrates the nonparametric flexibility of BART for individual‐level variable selection with a hierarchical spike‐and‐slab component for robust random‐effect selection. Recognizing that this unified model is hindered by near‐collinearity when cluster‐level covariates Z are constant within clusters, we further developed an innovative two‐step approach: first, we model individual‐level variability using random intercepts within the BART framework, and then we apply a permutation‐based selection method to rigorously assess the significance of cluster‐level predictors. To reduce the computational burden imposed by permutation, we also proposed the Sparse Dirichlet method, a lightweight approach that can be quickly applied for rapid screening. Together, these three methods provide researchers with a comprehensive and complementary toolkit for uncovering important patterns and relationships in high‐dimensional, multi‐site, or repeated‐measure healthcare data.

Our comprehensive simulation study, organized as a 2×2×2 factorial design crossing cluster structure (balanced vs. unbalanced), fixed‐effects specification (nonlinear vs. linear), and cluster‐level covariate structure (variant vs. constant Z), yielded several important findings. First, the Unified model achieved perfect random‐effect selection when Z varied within clusters, with $F_{1}\geq 0.85$ in every variant‐Z scenario and perfect selection in balanced settings, consistently outperforming PQL (which was competitive only when the fixed‐effects specification was linear). The Sparse Dirichlet has the similar performance and can be used as a competitive and computationally cheaper alternative in the unbalanced variant settings. This confirms that within‐cluster variation provides sufficient identifying information for joint selection, and that the Unified model is the clear choice in this setting. Second, the Two‐Step model proved to be the most robust approach when Z was constant within clusters, achieving random‐effect $F_{1}$ scores of 0.88 in both balanced constant‐Z scenarios and $F_{1}=0.81$ in the unbalanced nonlinear constant setting, consistently outperforming the Unified model, PQL, and the Sparse Dirichlet prior in these settings. Third, the inclusion of linear fixed‐effects scenarios, where BART's nonparametric flexibility confers no structural advantage, demonstrated that the proposed methods remain competitive even when parametric methods are correctly specified, alleviating concerns that BART‐based selection may be limited to nonlinear settings. Fourth, the unbalanced cluster design (K=35, $n_{k}\in \left\{10,\ldots ,30\right\}$), which mirrors the structure of our case study, confirmed that the Two‐Step model retains meaningful selection performance under realistic small‐sample conditions. Although the most extreme configuration (unbalanced clusters with linear fixed effects and constant Z) highlighted the inherent difficulty of cluster‐level identification when both the fixed‐effects surface is simple and the clusters are small, the modularity of the two‐step framework provided a solution: by substituting a correctly specified Bayesian linear mixed model in Step 1, the Two‐Step (Bayesian‐Linear) variant achieved $F_{1}=0.77$, demonstrating that researchers can adapt the framework to the expected data‐generating mechanism.

The sensitivity analyses further strengthened these conclusions. Random‐effect selection by the Two‐Step (MI) method was remarkably insensitive to both increasing individual‐level dimensionality (P=10 vs. P=50, with $F_{1}$ differences ≤0.01) and varying residual noise ($\sigma_{\varepsilon }\in \left\{0.1,0.5,1.0\right\}$, with $F_{1}$ stable at 0.87–0.89 throughout). This stability is a direct consequence of the Two‐Step architecture: because Step 2 operates on the K‐dimensional vector of estimated random intercepts rather than the full P‐dimensional covariate set, increasing P does not propagate into the random‐effect selection stage. The permutation threshold analysis confirmed that α=0.05 provides a well‐balanced trade‐off between Precision and Recall, with $F_{1}$ improving steadily from 0.82 at α=0.005 to 0.90 at α=0.1. The noise sensitivity analysis with varying numbers of noise random predictors (3 to 6) also demonstrated that the Two‐Step model maintained stable $F_{1}$ scores (0.88–0.90) across all noise levels, with MI consistently emerging as the best‐performing importance criterion.

The practical utility of our methods was demonstrated through the case study linking the Pathways study with the NJ SDOH database. Across both ZIP‐code and city‐level clustering structures, BMI and VO_2_max were the most frequently selected individual‐level predictors, consistent with prior literature on its association with cognitive performance in older adults. At the cluster level, the two‐step model identified socioeconomic variables—the proportion of adults aged 25+ with only a high‐school diploma at the ZIP‐code level and median household income and the percentage of population with a disability at the city level—that are plausibly linked to cognitive health through access to resources and community infrastructure. The unified model the proportion of adults aged 25+ with only a high‐school diploma (city level), while PQL failed to select any cluster‐level variables in either analysis. Predictive performance (elpd_loo) was comparable across the BART‐based models, with PQL consistently underperforming. Although the predictive differences among our methods were small and not statistically significant, we emphasize that the primary contribution of our framework is *multilevel variable selection* with explicit error control via permutation calibration, rather than maximizing black‐box predictive accuracy. In many biomedical and public‐health settings, the scientific objective is to identify interpretable drivers of within‐cluster outcomes and between‐cluster heterogeneity, where correctly recovering signal variables and limiting false discoveries is more valuable than marginal improvements in out‐of‐sample prediction.

It is important to recognize the assumptions underlying the classical random‐effects framework. Standard mixed‐effects models generally assume that the random effects are independent of the fixed effects and of the covariates used to parameterize them. This assumption may be violated in practice. In our simulation setting with cluster‐constant covariates, for example, the random effects β depend on Z. When this occurs, the unified model is misspecified, which can reduce the efficiency of variable selection by obscuring the distinction between variation explained by the random effects and that explained by the fixed effects, particularly for cluster‐level covariates. The Two‐Step approach is less affected because its cluster‐level selection stage does not rely on independence between the random effects and Z.

Based on our findings, we offer the following practical guidance for applied researchers. When cluster‐level covariates vary within clusters, the Unified model is the recommended approach, as it leverages within‐cluster variation for accurate joint selection. When cluster‐level covariates are constant within clusters—a common scenario in health services research where geographic or institutional indicators define clusters—the Two‐Step model should be preferred, as it consistently outperformed all alternatives in this setting. For rapid preliminary screening or computationally constrained settings, the Sparse Dirichlet prior offers a practical first pass. PQL may serve as a computationally efficient baseline for correctly specified linear models with variant Z, but is not recommended when nonlinear relationships are suspected or when cluster‐level covariates are constant.

There are several promising directions for future research. First, our methods currently focus on continuous outcomes; extending them to handle binary, multinomial, or ordinal outcomes would broaden their applicability to a wider range of healthcare datasets. Second, while we adopt BART for fixed effects, our current random effects specification remains linear, modeling them as a linear combination of $Z_{\mathrm{ik}}$ and $\beta_{k}$. A natural extension is to improve the flexibility of the random effects component by replacing the linear form $Z_{\mathrm{ik}}\beta_{k}$ with a nonparametric structure, such as a Gaussian Process with spike‐and‐slab priors [42], to better capture complex, non‐linear relationships and uncover subtle cluster‐level signals that may be missed by linear models. Finally, missing data remains a significant challenge in variable selection, particularly under the missing‐at‐random and missing‐not‐at‐random assumptions. Incorporating Bayesian group‐regularization priors into multiple imputation frameworks [43] may help improve selection stability in the presence of incomplete data. Together, these directions highlight the potential for further enhancing the flexibility, robustness, and practical utility of variable selection methods in mixed‐effects models.

## Funding

This work was supported by National Institutes of Health, 1R01HL159 077; Patient‐Centered Outcomes Research Institute, ME‐2021C2‐23685.

## Conflicts of Interest

The authors declare no conflicts of interest.

## Supporting information


**Table S1.** Performance metrics (mean ± SD across 250 replicates) for the four methods across all eight scenarios, with 3 useful and 3 noise **Z**. “Bal” is for “Balanced” setting, “Unbal” is for “Unbalanced”, “L” is for “Linear”. “NL” is for “Nonlinear”, “Var” is for “Variant” and “Con” is for “Constant”.
**Table S2.** Random‐effect selection performance for VIP, VIP Type, and MI criteria under varying numbers of noise random predictors, Balanced Nonlinear Constant Scenario. All settings use 3 useful Z.
**Table S3.** Alpha sensitivity analysis for random‐effect selection performance of the Two‐Step model (VIP, VIP Type, and MI criteria), Balanced Nonlinear Constant Scenario, with 3 useful and 3 noise Z.
**Table S4.** Random‐effect selection performance for VIP, VIP Type, and MI criteria of the Two‐Step model under varying P and σε, Balanced Nonlinear Constant Scenario, with 3 useful and 3 noise Z.
**Figure S1.** Performance metrics of two‐step method using MI as the number of noise covariates of cluster‐lever covariates Z increases from 3 to 6 while keeping the useful Z to fixed 3. The definition of three evaluation metrics are introduced in section 3.2.
**Figure S2.** F1 score of two‐step method using MI with the number of noise covariates of clusterlever covariates **Z** to 3 and the useful **Z** to 3 as the fixed effects total dimension varies 10 and 50 also with the variation of noise variance.
**Figure S3.** Sensitivity analysis of model performance across varying α levels (0.005 to 0.1) under four simulation scenarios of different **Z** using two‐step method with MI: 3 U, 3 N (3 useful **Z**, 3 noise **Z**); 3 U, 4 N (3 useful **Z**, 4 noise **Z**); 3 U, 5 N (3 useful **Z**, 5 noise **Z**); and 3 U, 6 N (3 useful **Z**, 6 noise **Z**). Each subfigure shows the metric trends: (a) F1, (b) Precision, (c) Recall, and (d) Type I error. The definition of the four evaluation metrics are introduced in section 3.2. Distinct line types and symbols are used to represent different simulation settings.

## Data Availability

R codes to implement our proposed methods and the comparison methods, and to replicate our simulation studies are provided in the GitHub page of the first author https://github.com/gosicksky/VS‐BART. Access to the case study data needs to be requested and approved by Rutgers University.

## References

[1] L. Breiman , “Random forests,” Machine Learning 45, no. 1 (2001): 5–32.

[2] T. Chen and C. Guestrin , “XGBoost: A Scalable Tree Boosting System,” *Proceedings of the 22nd ACM SIGKDD International Conference on Knowledge Discovery and Data Mining*, (2016), 785–794.

[3] H. A. Chipman , E. I. George , and R. E. McCulloch , “BART: Bayesian Additive Regression Trees,” Annals of Applied Statistics 4, no. 1 (2010): 266–298.

[4] J. Bleich , A. Kapelner , E. I. George , and S. T. Jensen , “Variable Selection for BART: An Application to Gene Regulation,” Annals of Applied Statistics 8, no. 3 (2014): 1750–1781.

[5] L. Hu , J. Y. Joyce Lin , and J. Ji , “Variable Selection With Missing Data in Both Covariates and Outcomes: Imputation and Machine Learning,” Statistical Methods in Medical Research 30, no. 12 (2021): 2651–2671. 34696650 10.1177/09622802211046385 PMC11181487

[6] J. Y. J. Lin , L. Hu , C. Huang , J. Jiayi , S. Lawrence , and U. Govindarajulu , “A Flexible Approach for Variable Selection in Large‐Scale Healthcare Database Studies With Missing Covariate and Outcome Data,” BMC Medical Research Methodology 22, no. 1 (2022): 132. 35508974 10.1186/s12874-022-01608-7 PMC9066834

[7] L. Hu , “A New Method for Clustered Survival Data: Estimation of Treatment Effect Heterogeneity and Variable Selection,” Biometrical Journal 66, no. 1 (2024): 2200178. 10.1002/bimj.202200178 PMC10953775 38072661

[8] C. Luo and M. J. Daniels , “Variable Selection Using Bayesian Additive Regression Trees,” Statistical Science 39, no. 2 (2024): 286–304. 39281973 10.1214/23-sts900 PMC11395240

[9] C. Spanbauer and R. Sparapani , “Nonparametric Machine Learning for Precision Medicine With Longitudinal Clinical Trials and Bayesian Additive Regression Trees With Mixed Models,” Statistics in Medicine 40, no. 11 (2021): 2665–2691. 33751659 10.1002/sim.8924

[10] B. Wundervald , A. Parnell , and K. Domijan , “Hierarchical Embedded Bayesian Additive Regression Trees,” arXiv preprint, 2022.

[11] L. Hu , J. Ji , R. D. Ennis , and J. W. Hogan , “A Flexible Approach for Causal Inference With Multiple Treatments and Clustered Survival Outcomes,” Statistics in Medicine 41, no. 25 (2022): 4982–4999. 35948011 10.1002/sim.9548 PMC9588538

[12] H. D. Bondell , A. Krishna , and S. K. Ghosh , “Joint Variable Selection for Fixed and Random Effects in Linear Mixed‐Effects Models,” Biometrics 66, no. 4 (2010): 1069–1077. 20163404 10.1111/j.1541-0420.2010.01391.x PMC2895687

[13] H. Peng and Y. Lu , “Model Selection in Linear Mixed Effect Models,” Journal of Multivariate Analysis 109 (2012): 109–129.

[14] F. K. Hui , S. Müller , and A. Welsh , “Joint Selection in Mixed Models Using Regularized PQL,” Journal of the American Statistical Association 112, no. 519 (2017): 1323–1333.

[15] Z. Chen and D. B. Dunson , “Random Effects Selection in Linear Mixed Models,” Biometrics 59, no. 4 (2003): 762–769. 14969453 10.1111/j.0006-341x.2003.00089.x

[16] S. Frühwirth‐Schnatter and H. Wagner , Bayesian Variable Selection for Random Intercept Modeling of Gaussian and Non‐Gaussian Data (Oxford University Press, 2010).

[17] F. Scheipl , “spikeSlabGAM: Bayesian Variable Selection, Model Choice and Regularization for Generalized Additive Mixed Models in R,” Journal of Statistical Software 43, no. 14 (2011): 1–24.

[18] A. R. Linero , “Bayesian Regression Trees for High‐Dimensional Prediction and Variable Selection,” Journal of the American Statistical Association 113, no. 522 (2018): 626–636.

[19] H. A. Chipman , E. I. George , and E. Robert , “Bayesian CART Model Search,” Journal of the American Statistical Association 93, no. 443 (1998): 935–948.

[20] M. T. Pratola , “Efficient Metropolis–Hastings Proposal Mechanisms for Bayesian Regression Tree Models,” Bayesian Analysis 11, no. 3 (2016): 885–911.

[21] C. N. Joyner , C. S. McMahan , J. M. Tebbs , and C. R. Bilder , “From Mixed Effects Modeling to Spike and Slab Variable Selection: A Bayesian Regression Model for Group Testing Data,” Biometrics 76, no. 3 (2020): 913–923. 31729015 10.1111/biom.13176 PMC7944974

[22] R. Sparapani , C. Spanbauer , and R. McCulloch , “Nonparametric Machine Learning and Efficient Computation With Bayesian Additive Regression Trees: The BART R Package,” Journal of Statistical Software 97, no. 1 (2021): 1–66.

[23] Y. V. Tan and J. Roy , “Bayesian Additive Regression Trees and the General BART Model,” Statistics in Medicine 38, no. 25 (2019): 5048–5069. 31460678 10.1002/sim.8347 PMC6800811

[24] D. A. Van Dyk and T. Park , “Partially Collapsed Gibbs Samplers: Theory and Methods,” Journal of the American Statistical Association 103, no. 482 (2008): 790–796.

[25] A. Groll and G. Tutz , “Variable Selection for Generalized Linear Mixed Models by L1‐Penalized Estimation,” Statistics and Computing 20, no. 2 (2010): 205–220.

[26] A. Hajjem , F. Bellavance , and D. Larocque , “Mixed‐Effects Random Forest for Clustered Data,” Journal of Statistical Computation and Simulation 84, no. 6 (2014): 1313–1328.

[27] R. J. Sela and J. S. Simonoff , “RE‐EM Trees: A Data Mining Approach for Longitudinal and Clustered Data,” Machine Learning 86, no. 2 (2012): 169–207.

[28] P. C. Bürkner , “brms: An R Package for Bayesian Multilevel Models Using Stan,” Journal of Statistical Software 80, no. 1 (2017): 1–28.

[29] P. C. Bürkner , “Advanced Bayesian Multilevel Modeling With the R Package brms,” R Journal 10, no. 1 (2018): 395–411.

[30] T. J. Mitchell and J. J. Beauchamp , “Bayesian Variable Selection in Linear Regression,” Journal of the American Statistical Association 83, no. 404 (1988): 1023–1032, 10.1080/01621459.1988.10478694.

[31] E. I. George and R. E. McCulloch , “Variable Selection via Gibbs Sampling,” Journal of the American Statistical Association 88, no. 423 (1993): 881–889.

[32] E. I. George and R. E. McCulloch , “Approaches for Bayesian Variable Selection,” Statistica Sinica 7, no. 2 (1997): 339–373.

[33] N. Sinha , B. A. Fausto , B. Mander , and M. A. Gluck , “High‐Quality Sleep Mitigates ABCA7‐Related Generalization Deficits in Healthy Older African Americans,” Journal of Alzheimer's Disease 94, no. 1 (2023): 281–290. 10.3233/JAD-230043 PMC10357211 37212111

[34] Centers for Disease Control and Prevention , “Non‐Medical Factors That Affect Alzheimer's Disease and Related Dementias Risk,” National Center for Chronic Disease Prevention and Health Promotion, 2024, https://www.cdc.gov/alzheimers‐dementia/php/sdoh/index.html.

[35] M. Quartagno , S. Grund , and J. Carpenter , “Jomo: A Flexible Package for Two‐Level Joint Modelling Multiple Imputation,” R Journal 9, no. 1 (2019): 205–228.

[36] A. Vehtari , A. Gelman , and J. Gabry , “Practical Bayesian Model Evaluation Using Leave‐One‐Out Cross‐Validation and WAIC,” Statistics and Computing 27 (2017): 1413–1432.

[37] A. Vehtari , D. Simpson , A. Gelman , Y. Yao , and J. Gabry , “Pareto Smoothed Importance Sampling,” Journal of Machine Learning Research 25, no. 72 (2024): 1–58.41334350

[38] J. N. Moody , K. E. Valerio , A. N. Hasselbach , et al., “Body Mass Index and Polygenic Risk for Alzheimer's Disease Predict Conversion to Alzheimer's Disease,” Journals of Gerontology: Series A 76, no. 8 (2021): 1415–1422. 10.1093/gerona/glab117 PMC8277084 33880516

[39] S. Kurl , J. Laukkanen , E. Lonnroos , A. Remes , and H. Soininen , “Cardiorespiratory Fitness and Risk of Dementia: A Prospective Population‐Based Cohort Study,” Age and Ageing 47, no. 4 (2018): 611–614. 29718064 10.1093/ageing/afy060

[40] T. L. Michaud , M. Siahpush , P. A. Farazi , et al., “The Association Between Body Mass Index, and Cognitive, Functional, and Behavioral Declines for Incident Dementia,” Journal of Alzheimer's Disease 66, no. 4 (2018): 1507–1517. 10.3233/JAD-180278 PMC6441968 30412484

[41] D. Predovan , N. Berryman , M. Lussier , et al., “Assessment of the Relationship Between Executive Function and Cardiorespiratory Fitness in Healthy Older Adults,” Frontiers in Psychology 12 (2021): 742184. 34803824 10.3389/fpsyg.2021.742184 PMC8595132

[42] H. Dance and B. Paige , “Fast and Scalable Spike and Slab Variable Selection in High‐Dimensional Gaussian Processes,” 2022, https://arxiv.org/abs/2111.04558.

[43] J. Zou , S. Wang , and Q. Chen , “Bayesian MI‐LASSO for Variable Selection on Multiply‐Imputed Data,” 2022, https://arxiv.org/abs/2211.00114.

