# Supplementary material for: Multi‐Level Variable Selection Using a BART‐Enhanced Mixed‐Effects Framework
Source: Stat Med. 2026 May 18;45:e70593. doi: 10.1002/sim.70593 (PMC13183514; doi:10.1002/sim.70593)
Supplement: Supplementary file 1 — Table S1. Performance metrics (mean ± SD across 250 replicates) for the four methods across all eight scenarios, with 3 useful and 3 noise Z. “Bal” is for “Balanced” setting, “Unbal” is for “Unbalanced”, “L” is for “Linear”. “NL” is for “Nonlinear”, “Var” is for “Variant” and “Con” is for “Constant”. Table S2. Random‐effect selection performance for VIP, VIP Type, and MI criteria under varying numbers of noise random predictors, Balanced Nonlinear Constant Scenario. All settings use 3 useful Z. Table S3. Alpha sensitivity analysis for random‐effect selection performance of the Two‐Step model (VIP, VIP Type, and MI criteria), Balanced Nonlinear Constant Scenario, with 3 useful and 3 noise Z. Table S4. Random‐effect selection performance for VIP, VIP Type, and MI criteria of the Two‐Step model under varying P and σε, Balanced Nonlinear Constant Scenario, with 3 useful and 3 noise Z. Figure S1. Performance metrics of two‐step method using MI as the number of noise covariates of cluster‐lever covariates Z increases from 3 to 6 while keeping the useful Z to fixed 3. The definition of three evaluation metrics are introduced in section 3.2. Figure S2. F1 score of two‐step method using MI with the number of noise covariates of clusterlever covariates Z to 3 and the useful Z to 3 as the fixed effects total dimension varies 10 and 50 also with the variation of noise variance. Figure S3. Sensitivity analysis of model performance across varying α levels (0.005 to 0.1) under four simulation scenarios of different Z using two‐step method with MI: 3 U, 3 N (3 useful Z, 3 noise Z); 3 U, 4 N (3 useful Z, 4 noise Z); 3 U, 5 N (3 useful Z, 5 noise Z); and 3 U, 6 N (3 useful Z, 6 noise Z). Each subfigure shows the metric trends: (a) F1, (b) Precision, (c) Recall, and (d) Type I error. The definition of the four evaluation metrics are introduced in section 3.2. Distinct line types and symbols are used to represent different simulation settings. [file SIM-45-0-s001.pdf]

Supplementary Materials for "Multi-level variable selection using a BART-enhanced mixed-effects framework"

Keming Zhang[1], Yaoyao Li[2], Jungang Zou[3],
Sijian Wang[4], Bernadette Fausto[5], Liangyuan Hu*[2]

[1]Department of Biostatistics, Brown University, Providence, Rhode Island 02903, USA
[2]Department of Biostatistics and Epidemiology, Rutgers University, Piscataway, NJ 08854, USA
[3]Department of Biostatistics, Columbia University, New York, New York 10027, USA
[4]Department of Statistics, Rutgers University, Piscataway NJ, 08854 USA
[5]Center for Molecular and Behavioral Neuroscience, Rutgers University, Newark, NJ 07102, USA

A Algorithm of permutation-based variable selection for unified model

Algorithm 1 Permutation-based variable selection approach.

for $1 \leq s \leq L_{rep}$ **do**
  Run the Algorithm 1 on the original dataset $(\mathbf{y}, \mathbf{X}, \mathbf{Z})$, compute the variable importance scores $(v_{s,1}, v_{s,2}, \dots, v_{s,P})$ for $\mathbf{X}$ and submodel scores $(LPS_{s,submodel_1}, LPS_{s,submodel_2}, \dots, LPS_{s,submodel_{2Q}})$ for $\mathbf{Z}$
end for
Compute the average variable importance scores $\bar{v}_j = (\sum_{s=1}^{s=L_{rep}} v_{s,j})/L_{rep}$ for $j = 1, 2, \dots, P$ and average LPS scores model $L\bar{P}S_{submodel_g} = (\sum_{s=1}^{s=L_{rep}} LPS_{s,submodel_g})/L_{rep}$ for $g = 1, 2, \dots, 2^Q$
for $1 \leq r \leq L$ **do**
  Run the Algorithm 1 on the null dataset $(\mathbf{y}^*_{\mathbf{r}}, \mathbf{X}, \mathbf{Z})$, compute the variable importance scores $(v_{r,1}, v_{r,2}, \dots, v_{r,P})$ for $\mathbf{X}$ and submodel scores $(LPS_{r,submodel_1}, LPS_{r,submodel_2}, \dots, LPS_{r,submodel_{2Q}})$ for $\mathbf{Z}$
end for
for $1 \leq j \leq p$ **do**
  Compute the $1-\alpha$ quantile v_j^α of the empirical distribution $\sum_{r=1}^{L} \delta_{v^*_{r,j}}(\cdot)$ for the variable X_j
  if $\bar{v}_j > v_j^\alpha$ **then**
    Select X_j
  end if
end for
for $1 \leq g \leq 2^Q$ **do**
  Compute the $1-\alpha$ quantile $LPS^\alpha_{submodel_g}$ of the empirical distribution $\sum_{r=1}^{L} \delta_{LPS^*_{submodel_{r,g}}}(\cdot)$ for the gth submodel
  if $L\bar{P}S_{submodel_g} > LPS^\alpha_{submodel_g}$ **then**
    Select the gth submodel into the candidate set
  end if
end for
Choose the submodel that has the biggest $L\bar{P}S$ among the candidate set

B Derivation of posterior distributions

B.1 Conditional Sampling Distribution for b

Although $\mathbf{b}_k$ are latent variables, we still need to iterate their values in our algorithm as they represent the "random" component of the random effects. The prior of $\mathbf{b}_k$ is given by $\mathcal{MVN}(0, \mathbf{I})$. For each

iteration, we sample from the conditional distribution:

$$p(\mathbf{b}_k \mid -) = \mathcal{MVN}(\hat{\mathbf{h}}_k, \hat{\mathbf{H}}_k),$$

where $\hat{\mathbf{h}}_k = \sigma^{-2} \sum_{i=1}^{n_k} \mathbf{Z}_{ik} \mathbf{\Lambda} \mathbf{\Gamma} (y_{ik} - f(\mathbf{X}_{ik})) \hat{\mathbf{H}}_k$ and $\hat{\mathbf{H}}_k = (\sigma^{-2} \sum_{i=1}^{n_k} \mathbf{\Gamma}^\top \mathbf{\Lambda} \mathbf{Z}_{ik}^\top \mathbf{Z}_{ik} \mathbf{\Lambda} \mathbf{\Gamma} + \mathbf{I})^{-1}$.

B.2 Conditional Posterior for σ^2

The prior of σ^2 is given by inverse chi-square distribution $\sigma^2 \sim \nu\tau^2/\chi^2_\nu$. We have

$$p(\sigma^2 \mid -) = inv - \chi^2(\hat{\nu}, \hat{\tau^2}),$$

where $\hat{\nu} = \nu + \sum_{k=1}^K n_k$ and $\hat{\tau^2} = (\nu\tau^2 + \sum_{k=1}^K \sum_{i=1}^{n_k} (y_{ik} - f(\mathbf{X}_{ik}) - \mathbf{Z}_{ik}^\top \mathbf{\Lambda} \mathbf{\Gamma} \mathbf{b}_k)^2)/(\nu + \sum_{k=1}^K n_k)$.

B.3 Conditional Posterior for $\mathbf{\Gamma}$

We work on the transformed version of $\mathbf{\Gamma}$, the vector $\boldsymbol{\gamma}$. The prior of $\boldsymbol{\gamma}$ is given by $MVN(\boldsymbol{\gamma}_0, \mathbf{R}_0)$. Now let $\tilde{\mathbf{u}}_{ik} = (b_{kq}\lambda_u z_{iku} : q = 1, \dots, Q, u = l+1, \dots, Q)^\top$, which is a $\frac{Q(Q-1)}{2}$ dimensional vector, then

$$p(\boldsymbol{\gamma} \mid -) = MVN(\hat{\boldsymbol{\gamma}}, \hat{\mathbf{R}}),$$

where $\hat{\mathbf{R}} = \left(\sigma^{-2} \sum_{k=1}^K \sum_{i=1}^{n_k} \tilde{\mathbf{u}}_{ik} \tilde{\mathbf{u}}_{ik}^\top + \mathbf{R}_0^{-1}\right)^{-1}$ and $\hat{\boldsymbol{\gamma}} = \hat{\mathbf{R}} \left(\sigma^{-2} \sum_{k=1}^K \left(\sum_{i=1}^{n_k} \tilde{\mathbf{u}}_{ik} (y_{ik} - f(\mathbf{X}_{ik}) - \mathbf{Z}_k \mathbf{\Lambda} \mathbf{b}_k^T\right) + \mathbf{R}_0^{-1} \boldsymbol{\gamma}_0\right)$.

B.4 Conditional Posterior for $\mathbf{\Lambda}$, $\boldsymbol{v}$

Recall that $\mathbf{\Lambda}$ has Q elements that are assumed to be independent of each other. **?** thus suggested deriving the conditional distribution for each individual λ_q element for simplicity. The prior of λ_q is Dirac spike and slab prior. Let $\boldsymbol{\lambda}_{(-q)}$ be the vector of the $Q-1$ other elements of $\mathbf{\Lambda}$, except λ_q. Then

$$p(\lambda_q \mid -) = p(\lambda_q \mid v_q) \sim (1 - v_q)\delta_0 + v_q \mathcal{TN}(0, s_q^2), v_q \sim \text{Bernoulli}(\pi_q), \pi_q \sim \text{Beta}(c_q, d_q)$$

which is conditionally conjugate.

First we let $\tilde{t}_{ikq} = Z_{ikq} \left(b_{kq} + \sum_{u=1}^{q-1} b_{ku} \gamma_{qu}\right)$ for $q = 1, \dots, Q$. Let $\xi_{ikq} = y_{ik} - f(\mathbf{X}_{ik}) - \sum_{u \neq q} \tilde{t}_{iku} \lambda_u$ for each λ_q. The posterior for v_q is:

$$p(v_q \mid -) \propto p(v_q) \int p(\lambda_q \mid v_q) p(\mathbf{y} | \lambda_q, v_q, -) \, d\lambda_q.$$

For priors, $\lambda_q \sim \mathcal{TN}(\theta_q, s_q^2, (0, \infty))$ if $v_q = 1$, and $\lambda_q = 0$ if $v_q = 0$. We further define:

$$\mathcal{L}_1 = \int p(\lambda_q \mid v_q = 1) p(\mathbf{y} \mid \lambda_q, v_q = 1, -) \, d\lambda_q, \quad \mathcal{L}_0 = p(\mathbf{y} \mid v_q = 0, -) = \exp\left(-\frac{1}{2\sigma^2} \sum_{k=1}^K \sum_{i=1}^{n_k} \xi_{ikq}^2\right).$$

Among these components, the conditional likelihood $p(\mathbf{y} \mid \lambda_q, v_q = 1, -)$ is

$$p(\mathbf{y} \mid \lambda_q, v_q = 1, -) = \frac{\omega_q}{\sqrt{2\pi}} \exp\left(-\frac{\omega_q^2}{2} (\lambda_q - \tilde{\lambda}_q)^2\right).$$

where: $\omega_q^{-2} = \frac{1}{\sigma^2} \sum_{k=1}^K \sum_{i=1}^{n_k} \tilde{t}_{ikq}^2$, and $\tilde{\lambda}_q = \frac{\frac{1}{\sigma^2} \sum_{k=1}^K \sum_{i=1}^{n_k} \xi_{ikq} \tilde{t}_{ikq}}{\omega_q^{-2}}$. As defined before, the prior of λ_q when $v_q = 1$ is $p(\lambda_q \mid v_q = 1) = \frac{\frac{1}{\sqrt{2\pi s_q^2}} \exp\left(-\frac{1}{2s_q^2}(\lambda_q - \mu_q)^2\right)}{\Phi\left(\frac{\theta_q}{s_q}\right)}$. Then we can calculate $\mathcal{L}_1$:

$$\mathcal{L}_1 = \frac{\omega_q}{\sqrt{2\pi} \cdot \Phi\left(\frac{\theta_q}{s_q}\right)} \frac{\sqrt{2\pi \hat{s}_q^2}}{\sqrt{2\pi s_q^2}} \int_0^\infty \frac{1}{\sqrt{2\pi \hat{s}_q^2}} \exp\left(-\frac{(\lambda_q - \hat{\lambda}_q)^2}{2\hat{s}_q^2}\right) d\lambda_q \cdot \exp(\text{constant C}),$$

where constant $\mathrm{C} = \frac{\omega_q^2}{2}\tilde{\lambda}_q^2 + \frac{\theta_q^2}{2s_q^2} - \frac{\omega_q^4\tilde{\lambda}_q^2}{2\left(\omega_q^2+\frac{1}{s_q^2}\right)} - \frac{\omega_q^2\tilde{\lambda}_q\frac{\theta_q}{s_q^2}}{\omega_q^2+\frac{1}{s_q^2}} - \frac{\frac{\theta_q^2}{s_q^4}}{2\left(\omega_q^2+\frac{1}{s_q^2}\right)}$ and

$$\begin{aligned}\hat{s}_q^2 &= \left(\omega_q^2 + \frac{1}{s_q^2}\right)^{-1} \\ \hat{\lambda}_q &= \hat{s}_q^2\left(\omega_q^2\tilde{\lambda}_q + \frac{\theta_q}{s_q^2}\right)\end{aligned} \tag{1}$$

Noted that given the sampled value of v_q, the full conditional posterior distribution of λ_q is:

$$\lambda_q \mid - \sim \begin{cases}\delta_0, & \text{if } v_q = 0 \\ \mathcal{TN}(\hat{\lambda}_q, \hat{s}_q^2, (0,\infty)), & \text{if } v_q = 1\end{cases}$$

This implies that when $v_q = 0$, λ_q is deterministically set to 0 (the spike). When $v_q = 1$, λ_q is drawn from a truncated normal distribution centered at $\hat{\lambda}_q$ with variance $\hat{s}_q^2$ on the domain $(0,\infty)$.

Finally we can calculate and summarize $\mathcal{L}_0$ and $\mathcal{L}_1$:

$$\begin{aligned}\mathcal{L}_1 &= \frac{\omega_q}{\Phi\left(\frac{\mu_l}{s_q}\right)}\frac{\hat{s}_l}{s_q} \cdot \Phi\left(\frac{\hat{\lambda}_l}{\sqrt{\hat{s}_l^2}}\right) \cdot \exp\left(\frac{\omega_q^2}{2}\tilde{\lambda}_q^2 + \frac{\mu_l^2}{2s_q^2} - \frac{\omega_q^4\tilde{\lambda}_q^2}{2\left(\omega_q^2+\frac{1}{s_q^2}\right)} - \frac{\omega_q^2\tilde{\lambda}_q\frac{\mu_l}{s_q^2}}{\omega_q^2+\frac{1}{s_q^2}} - \frac{\frac{\mu_l^2}{s_q^4}}{2\left(\omega_q^2+\frac{1}{s_q^2}\right)}\right) \\ \mathcal{L}_0 &= p(\mathbf{y} \mid v_q = 0, -) = \exp\left(-\frac{1}{2\sigma^2}\sum_{k=1}^{K}\sum_{i=1}^{n_k}\xi_{ikq}^2\right)\end{aligned} \tag{2}$$

And the posterior probability for v_q is:

$$p(v_q = 1 \mid -) = \frac{\pi_q\mathcal{L}_1}{\pi_q\mathcal{L}_1 + (1-\pi_q)\mathcal{L}_0}.$$

B.5 Conditional Posterior for π_q

The prior for $\pi_q \sim \mathrm{Beta}(c_q, d_q)$, $q = 1, \ldots, Q$. The posterior is:

$$p(\pi_q \mid -) \propto p(\pi_q)p(v_q|\pi_q),$$

which simplifies to:

$$p(\pi_q \mid -) \propto \pi_q^{c_q+v_q-1}(1-\pi_q)^{d_q-v_q}.$$

Thus, the posterior is:

$$\pi_q \mid - \sim \mathrm{Beta}\left(c_q + v_q, d_q + 1 - v_q\right).$$

C Full Simulation Results

The simulation results of balanced and unbalanced are in Web Table 1.

Web Table 1. Performance metrics (mean ± SD across 250 replicates) for the four methods across all eight scenarios, with 3 useful and 3 noise **Z**. "Bal" is for "Balanced" setting, "Unbal" is for "Unbalanced", "L" is for "Linear". "NL" is for "Nonlinear", "Var" is for "Variant" and "Con" is for "Constant".

Scenario	Method	Fixed Effect			Random Effect			
		Recall (SD)	Precision (SD)	F_1 (SD)	Recall (SD)	Precision (SD)	F_1 (SD)	Type I error (SD)
Balanced design ($K = 50$, $n_k = 100$, $N = 5{,}000$)								
Bal–NL–Var	Unified (VIP)	0.80 (0.00)	1.00 (0.00)	0.89 (0.00)				
	Unified (VIP Type)	0.96 (0.11)	1.00 (0.00)	0.95 (0.06)	1.00 (0.00)	1.00 (0.00)	1.00 (0.00)	0.00 (0.00)
	Unified (MI)	0.90 (0.18)	0.99 (0.04)	0.94 (0.11)				
	Two-Step (MI)		—				—	
	Sparse	0.83 (0.07)	1.00 (0.03)	0.90 (0.03)	1.00 (0.00)	1.00 (0.02)	1.00 (0.01)	0.00 (0.02)
	PQL	0.87 (0.09)	0.80 (0.15)	0.82 (0.09)	0.94 (0.17)	1.00 (0.03)	0.96 (0.12)	0.00 (0.00)
Bal–NL–Con	Unified (VIP)	0.80 (0.00)	1.00 (0.00)	0.89 (0.00)				
	Unified (VIP Type)	0.87 (0.12)	1.00 (0.00)	0.92 (0.07)	0.73 (0.24)	0.59 (0.19)	0.64 (0.19)	0.56 (0.31)
	Unified (MI)	0.85 (0.22)	1.00 (0.02)	0.91 (0.14)				
	Two-Step (VIP)	0.80 (0.00)	1.00 (0.00)	0.89 (0.00)	0.73 (0.22)	0.99 (0.05)	0.83 (0.16)	0.07 (0.13)
	Two-Step (VIP Type)	0.87 (0.12)	1.00 (0.00)	0.92 (0.07)	0.60 (0.14)	0.99 (0.08)	0.74 (0.13)	0.05 (0.14)
	Two-Step (MI)	0.85 (0.22)	1.00 (0.02)	0.91 (0.14)	0.85 (0.23)	0.96 (0.14)	0.88 (0.18)	0.04 (0.11)
	Sparse	0.82 (0.07)	0.99 (0.04)	0.90 (0.03)	0.70 (0.25)	0.69 (0.23)	0.66 (0.18)	0.40 (0.33)
	PQL	0.80 (0.01)	1.00 (0.01)	0.89 (0.01)	0.47 (0.22)	0.98 (0.08)	0.61 (0.17)	0.02 (0.11)
Bal–L–Var	Unified (VIP)	0.75 (0.00)	1.00 (0.00)	0.86 (0.00)				
	Unified (VIP Type)	0.97 (0.09)	1.00 (0.00)	0.98 (0.05)	1.00 (0.00)	1.00 (0.00)	1.00 (0.00)	0.00 (0.00)
	Unified (MI)	1.00 (0.00)	0.99 (0.04)	1.00 (0.02)				
	Two-Step (MI)		—				—	
	Sparse	0.94 (0.11)	0.97 (0.08)	0.95 (0.07)	1.00 (0.00)	1.00 (0.00)	1.00 (0.00)	0.00 (0.00)
	PQL	1.00 (0.00)	0.85 (0.15)	0.91 (0.09)	1.00 (0.00)	1.00 (0.00)	1.00 (0.00)	0.00 (0.00)
Bal–L–Con	Unified (VIP)	0.75 (0.00)	1.00 (0.00)	0.86 (0.00)				
	Unified (VIP Type)	0.95 (0.10)	1.00 (0.00)	0.97 (0.06)	0.76 (0.25)	0.59 (0.19)	0.64 (0.19)	0.57 (0.32)
	Unified (MI)	1.00 (0.00)	1.00 (0.03)	1.00 (0.02)				
	Two-Step (VIP)	0.75 (0.00)	1.00 (0.00)	0.86 (0.00)	0.73 (0.22)	0.99 (0.07)	0.82 (0.17)	0.07 (0.12)
	Two-Step (VIP Type)	0.95 (0.10)	1.00 (0.00)	0.97 (0.06)	0.59 (0.14)	0.99 (0.08)	0.73 (0.13)	0.03 (0.15)
	Two-Step (MI)	1.00 (0.00)	1.00 (0.03)	1.00 (0.02)	0.84 (0.22)	0.95 (0.14)	0.88 (0.19)	0.04 (0.11)
	Sparse	0.94 (0.11)	0.97 (0.07)	0.95 (0.07)	0.68 (0.25)	0.69 (0.23)	0.65 (0.19)	0.38 (0.33)
	PQL	1.00 (0.00)	1.00 (0.00)	1.00 (0.00)	0.46 (0.20)	0.98 (0.09)	0.60 (0.16)	0.02 (0.08)
Unbalanced design ($K = 35$, $n_k \sim$ Uniform$\{10, \ldots, 30\}$, $N = 648$)								
Unbal–NL–Var	Unified (VIP)	0.80 (0.00)	1.00 (0.00)	0.89 (0.00)				
	Unified (VIP Type)	0.84 (0.12)	1.00 (0.00)	0.91 (0.08)	0.78 (0.25)	1.00 (0.02)	0.85 (0.20)	0.00 (0.02)
	Unified (MI)	0.84 (0.08)	0.99 (0.03)	0.91 (0.05)				
	Two-Step (MI)		—				—	
	Sparse	1.00 (0.01)	0.98 (0.06)	0.99 (0.04)	0.79 (0.25)	1.00 (0.04)	0.87 (0.15)	0.01 (0.04)
	PQL	0.82 (0.10)	0.85 (0.18)	0.82 (0.10)	0.80 (0.27)	0.91 (0.17)	0.80 (0.19)	0.16 (0.31)
Unbal–NL–Con	Unified (VIP)	0.80 (0.00)	1.00 (0.00)	0.89 (0.00)				
	Unified (VIP Type)	1.00 (0.03)	1.00 (0.00)	1.00 (0.02)	0.33 (0.00)	0.99 (0.08)	0.50 (0.02)	0.00 (0.00)
	Unified (MI)	0.80 (0.00)	1.00 (0.00)	0.89 (0.00)				
	Two-Step (VIP)	0.80 (0.00)	1.00 (0.00)	0.89 (0.00)	0.52 (0.17)	1.00 (0.00)	0.67 (0.15)	0.08 (0.07)
	Two-Step (VIP Type)	1.00 (0.03)	1.00 (0.00)	1.00 (0.02)	0.33 (0.02)	1.00 (0.00)	0.50 (0.02)	0.07 (0.06)
	Two-Step (MI)	0.80 (0.00)	1.00 (0.00)	0.89 (0.00)	0.68 (0.07)	0.98 (0.02)	0.81 (0.04)	0.01 (0.02)
	Sparse	1.00 (0.00)	0.98 (0.05)	0.99 (0.03)	0.56 (0.24)	0.78 (0.24)	0.61 (0.18)	0.24 (0.29)
	PQL	0.80 (0.00)	1.00 (0.00)	0.89 (0.00)	0.33 (0.00)	1.00 (0.00)	0.50 (0.00)	0.00 (0.00)
Unbal–L–Var	Unified (VIP)	0.75 (0.00)	1.00 (0.00)	0.86 (0.00)				
	Unified (VIP Type)	0.99 (0.06)	1.00 (0.00)	0.99 (0.03)	0.84 (0.23)	1.00 (0.02)	0.89 (0.18)	0.00 (0.02)
	Unified (MI)	0.97 (0.08)	0.97 (0.07)	0.97 (0.06)				
	Two-Step (MI)		—				—	
	Sparse	1.00 (0.00)	0.99 (0.05)	1.00 (0.03)	0.85 (0.22)	1.00 (0.02)	0.91 (0.14)	0.00 (0.03)
	PQL	1.00 (0.00)	0.64 (0.17)	0.77 (0.12)	0.93 (0.16)	0.93 (0.14)	0.92 (0.12)	0.10 (0.23)
Unbal–L–Con	Unified (VIP)	0.75 (0.00)	1.00 (0.00)	0.86 (0.00)				
	Unified (VIP Type)	0.80 (0.10)	1.00 (0.00)	0.89 (0.06)	0.68 (0.26)	0.70 (0.24)	0.67 (0.22)	0.34 (0.29)
	Unified (MI)	0.97 (0.08)	0.99 (0.05)	0.98 (0.05)				
	Two-Step (VIP)	0.75 (0.00)	1.00 (0.00)	0.86 (0.00)	0.46 (0.25)	0.88 (0.32)	0.58 (0.26)	0.05 (0.13)
	Two-Step (VIP Type)	0.80 (0.10)	1.00 (0.00)	0.89 (0.06)	0.34 (0.22)	0.79 (0.40)	0.46 (0.26)	0.06 (0.18)
	Two-Step (MI)	0.97 (0.08)	0.99 (0.05)	0.98 (0.05)	0.53 (0.31)	0.79 (0.37)	0.62 (0.32)	0.06 (0.13)
	Sparse	1.00 (0.00)	0.98 (0.07)	0.99 (0.04)	0.56 (0.23)	0.79 (0.24)	0.62 (0.18)	0.23 (0.29)
	PQL	1.00 (0.00)	1.00 (0.01)	1.00 (0.01)	0.48 (0.22)	0.94 (0.16)	0.61 (0.16)	0.07 (0.19)

Note. The balanced and unbalanced designs differ substantially in total sample size ($N = 5{,}000$ vs. $N = 648$). Cross-panel comparisons therefore reflect the joint effect of cluster imbalance *and* reduced sample size.

Web Table 2. Random-effect selection performance for VIP, VIP Type, and MI criteria under varying numbers of noise random predictors, Balanced Nonlinear Constant Scenario. All settings use 3 useful **Z**.

Scenario	Methods	Fixed Effect			Random Effect			
		Recall (SD)	Precision (SD)	F_1 (SD)	Recall (SD)	Precision (SD)	F_1 (SD)	Type I error (SD)
3 Useful **Z**, 3 Noise **Z**	PQL	0.80 (0.01)	1.00 (0.01)	0.89 (0.01)	0.45 (0.22)	0.98 (0.08)	0.61 (0.17)	0.02 (0.11)
	Unified (VIP)	0.80 (0.00)	1.00 (0.00)	0.89 (0.00)				
	Unified (VIP Type)	0.87 (0.12)	1.00 (0.00)	0.92 (0.07)	0.73 (0.24)	0.59 (0.19)	0.64 (0.19)	0.56 (0.31)
	Unified (MI)	0.85 (0.22)	1.00 (0.02)	0.91 (0.14)				
	Sparse	0.82 (0.07)	0.99 (0.04)	0.90 (0.03)	0.70 (0.25)	0.69 (0.23)	0.66 (0.18)	0.40 (0.33)
	Two-Step (VIP)	0.80 (0.00)	1.00 (0.00)	0.89 (0.00)	0.73 (0.22)	0.99 (0.05)	0.83 (0.16)	0.27 (0.22)
	Two-Step (VIP Type)	0.85 (0.22)	1.00 (0.02)	0.91 (0.14)	0.60 (0.14)	0.99 (0.08)	0.74 (0.13)	0.40 (0.14)
	Two-Step (MI)	0.85 (0.22)	1.00 (0.02)	0.91 (0.14)	0.85 (0.23)	0.96 (0.14)	0.88 (0.18)	0.18 (0.24)
3 Useful **Z**, 4 Noise **Z**	PQL	0.80 (0.00)	1.00 (0.01)	0.89 (0.01)	0.48 (0.21)	0.97 (0.11)	0.61 (0.17)	0.02 (0.08)
	Unified (VIP)	0.80 (0.00)	1.00 (0.00)	0.89 (0.00)				
	Unified (VIP Type)	0.88 (0.12)	1.00 (0.00)	0.93 (0.07)	0.70 (0.25)	0.55 (0.21)	0.60 (0.19)	0.47 (0.29)
	Unified (MI)	0.86 (0.22)	1.00 (0.02)	0.91 (0.14)				
	Sparse	0.83 (0.07)	0.99 (0.03)	0.90 (0.03)	0.67 (0.24)	0.49 (0.15)	0.56 (0.16)	0.31 (0.25)
	Two-Step (VIP)	0.80 (0.00)	1.00 (0.00)	0.89 (0.00)	0.81 (0.20)	0.99 (0.05)	0.87 (0.14)	0.20 (0.20)
	Two-Step (VIP Type)	0.88 (0.12)	1.00 (0.00)	0.93 (0.07)	0.61 (0.13)	0.98 (0.09)	0.75 (0.12)	0.39 (0.13)
	Two-Step (MI)	0.86 (0.22)	1.00 (0.02)	0.91 (0.14)	0.90 (0.19)	0.93 (0.13)	0.90 (0.14)	0.16 (0.20)
3 Useful **Z**, 5 Noise **Z**	PQL	0.80 (0.00)	1.00 (0.00)	0.89 (0.00)	0.47 (0.20)	0.95 (0.14)	0.60 (0.15)	0.03 (0.10)
	Unified (VIP)	0.80 (0.00)	1.00 (0.00)	0.89 (0.00)				
	Unified (VIP Type)	0.88 (0.12)	1.00 (0.00)	0.93 (0.07)	0.72 (0.26)	0.52 (0.21)	0.59 (0.20)	0.44 (0.27)
	Unified (MI)	0.86 (0.22)	1.00 (0.02)	0.91 (0.14)				
	Sparse	0.83 (0.07)	1.00 (0.03)	0.90 (0.03)	0.67 (0.26)	0.47 (0.15)	0.54 (0.17)	0.28 (0.22)
	Two-Step (VIP)	0.80 (0.00)	1.00 (0.00)	0.89 (0.00)	0.85 (0.21)	0.99 (0.08)	0.90 (0.15)	0.15 (0.21)
	Two-Step (VIP Type)	0.88 (0.12)	1.00 (0.00)	0.93 (0.07)	0.63 (0.11)	0.98 (0.09)	0.76 (0.10)	0.38 (0.11)
	Two-Step (MI)	0.86 (0.22)	1.00 (0.02)	0.91 (0.14)	0.91 (0.19)	0.90 (0.16)	0.89 (0.16)	0.16 (0.21)
3 Useful **Z**, 6 Noise **Z**	PQL	0.80 (0.00)	1.00 (0.00)	0.89 (0.00)	0.49 (0.21)	0.96 (0.13)	0.61 (0.16)	0.03 (0.08)
	Unified (VIP)	0.80 (0.00)	1.00 (0.00)	0.89 (0.00)				
	Unified (VIP Type)	0.90 (0.12)	1.00 (0.00)	0.94 (0.07)	0.65 (0.25)	0.50 (0.19)	0.52 (0.19)	0.37 (0.22)
	Unified (MI)	0.92 (0.17)	1.00 (0.02)	0.95 (0.12)				
	Sparse	0.83 (0.08)	0.99 (0.03)	0.91 (0.04)	0.69 (0.25)	0.44 (0.14)	0.52 (0.16)	0.32 (0.23)
	Two-Step (VIP)	0.80 (0.00)	1.00 (0.00)	0.89 (0.00)	0.87 (0.19)	0.98 (0.08)	0.91 (0.14)	0.13 (0.20)
	Two-Step (VIP Type)	0.90 (0.12)	1.00 (0.00)	0.94 (0.07)	0.63 (0.13)	0.96 (0.14)	0.75 (0.12)	0.39 (0.14)
	Two-Step (MI)	0.92 (0.17)	1.00 (0.02)	0.95 (0.12)	0.93 (0.17)	0.87 (0.16)	0.89 (0.15)	0.18 (0.20)

Web Table 3. Alpha sensitivity analysis for random-effect selection performance of the Two-Step model (VIP, VIP Type, and MI criteria), Balanced Nonlinear Constant Scenario, with 3 useful and 3 noise **Z**.

α	Method	Random Effect			
		Recall (SD)	Precision (SD)	F_1 (SD)	Type I error (SD)
0.005	Two-Step (VIP)	0.55 (0.25)	0.93 (0.25)	0.67 (0.24)	0.001 (0.02)
	Two-Step (VIP Type)	0.50 (0.19)	0.96 (0.20)	0.64 (0.20)	0.004 (0.04)
	Two-Step (MI)	0.78 (0.30)	0.92 (0.24)	0.82 (0.26)	0.02 (0.08)
0.01	Two-Step (VIP)	0.60 (0.23)	0.97 (0.16)	0.72 (0.20)	0.001 (0.02)
	Two-Step (VIP Type)	0.53 (0.18)	0.97 (0.16)	0.67 (0.18)	0.004 (0.04)
	Two-Step (MI)	0.80 (0.28)	0.94 (0.21)	0.84 (0.24)	0.02 (0.09)
0.05	Two-Step (VIP)	0.73 (0.22)	0.99 (0.05)	0.83 (0.16)	0.01 (0.04)
	Two-Step (VIP Type)	0.60 (0.14)	0.99 (0.08)	0.74 (0.13)	0.01 (0.05)
	Two-Step (MI)	0.85 (0.23)	0.96 (0.14)	0.88 (0.18)	0.04 (0.11)
0.1	Two-Step (VIP)	0.80 (0.20)	0.99 (0.06)	0.87 (0.14)	0.01 (0.05)
	Two-Step (VIP Type)	0.61 (0.12)	0.99 (0.09)	0.75 (0.12)	0.01 (0.06)
	Two-Step (MI)	0.88 (0.20)	0.95 (0.11)	0.90 (0.15)	0.05 (0.12)

Web Table 4. Random-effect selection performance for VIP, VIP Type, and MI criteria of the Two-Step model under varying P and σ_ε, Balanced Nonlinear Constant Scenario, with 3 useful and 3 noise **Z**.

P	σ_ε	**Method**	**Random Effect**			
			Recall (SD)	Precision (SD)	F_1 (SD)	Type I error (SD)
10	0.1	Two-Step (VIP)	0.72 (0.23)	0.98 (0.12)	0.81 (0.18)	0.01 (0.05)
		Two-Step (VIP Type)	0.59 (0.15)	0.98 (0.11)	0.73 (0.14)	0.01 (0.05)
		Two-Step (MI)	0.85 (0.22)	0.95 (0.14)	0.88 (0.17)	0.04 (0.11)
	0.5	Two-Step (VIP)	0.73 (0.23)	0.99 (0.10)	0.82 (0.18)	0.01 (0.05)
		Two-Step (VIP Type)	0.59 (0.15)	0.99 (0.10)	0.73 (0.14)	0.01 (0.05)
		Two-Step (MI)	0.86 (0.22)	0.95 (0.15)	0.89 (0.18)	0.04 (0.11)
	1.0	Two-Step (VIP)	0.73 (0.22)	0.99 (0.05)	0.83 (0.16)	0.01 (0.05)
		Two-Step (VIP Type)	0.60 (0.14)	0.99 (0.08)	0.74 (0.13)	0.01 (0.05)
		Two-Step (MI)	0.85 (0.23)	0.96 (0.14)	0.88 (0.18)	0.04 (0.11)
50	0.1	Two-Step (VIP)	0.71 (0.24)	0.99 (0.10)	0.81 (0.18)	0.01 (0.05)
		Two-Step (VIP Type)	0.58 (0.15)	0.99 (0.08)	0.72 (0.14)	0.01 (0.05)
		Two-Step (MI)	0.84 (0.23)	0.95 (0.16)	0.87 (0.19)	0.04 (0.11)
	0.5	Two-Step (VIP)	0.71 (0.23)	0.98 (0.11)	0.81 (0.18)	0.01 (0.04)
		Two-Step (VIP Type)	0.58 (0.15)	0.98 (0.11)	0.72 (0.15)	0.01 (0.05)
		Two-Step (MI)	0.84 (0.23)	0.95 (0.16)	0.88 (0.19)	0.04 (0.11)
	1.0	Two-Step (VIP)	0.71 (0.24)	0.99 (0.11)	0.80 (0.18)	0.002 (0.05)
		Two-Step (VIP Type)	0.59 (0.15)	0.98 (0.12)	0.72 (0.15)	0.004 (0.04)
		Two-Step (MI)	0.84 (0.24)	0.94 (0.18)	0.87 (0.20)	0.04 (0.11)

D Figures

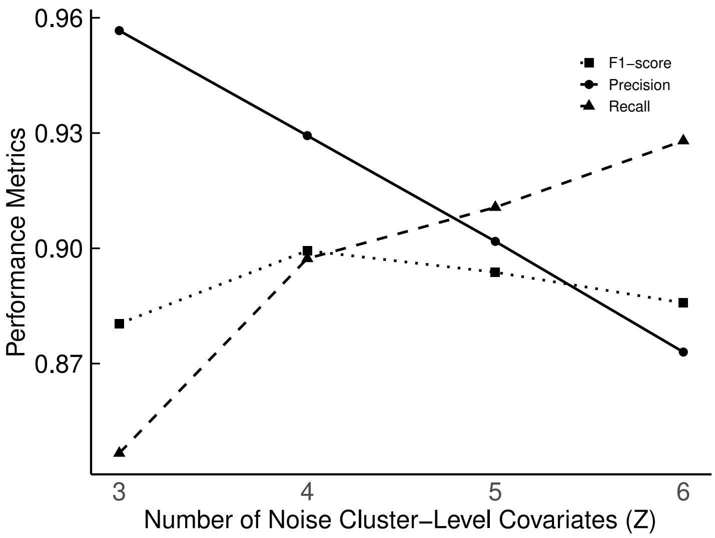

Web Figure 1. Performance metrics of two-step method using MI as the number of noise covariates of cluster-lever covariates **Z** increases from 3 to 6 while keeping the useful **Z** to fixed 3. The definition of three evaluation metrics are introduced in section 3.2.

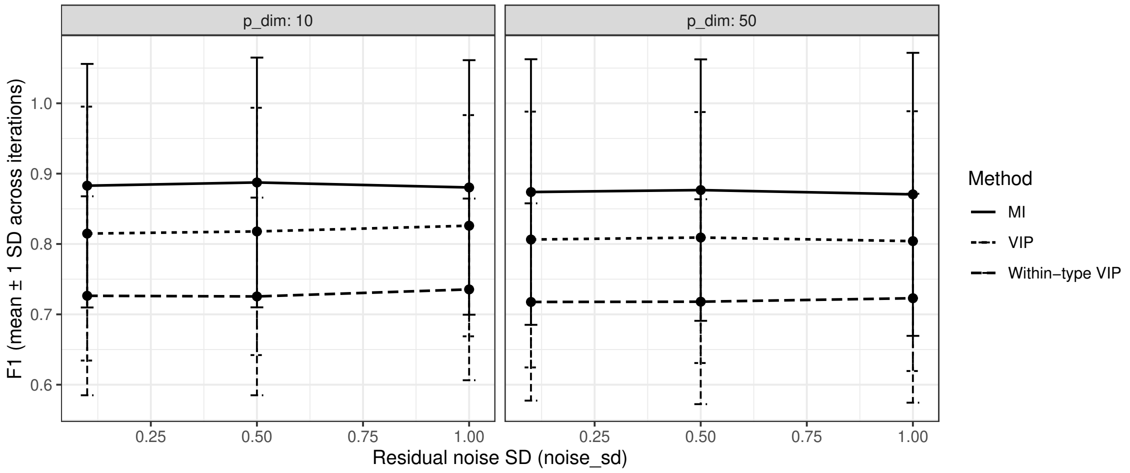

Web Figure 2. F1 score of two-step method using MI with the number of noise covariates of cluster-lever covariates **Z** to 3 and the useful **Z** to 3 as the fixed effects total dimension varies 10 and 50 also with the variation of noise variance.

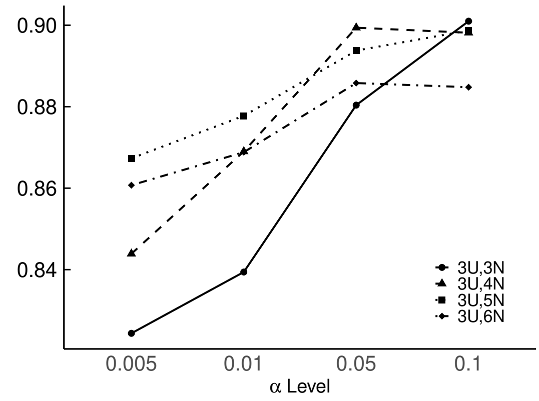

(a) F_1 scores

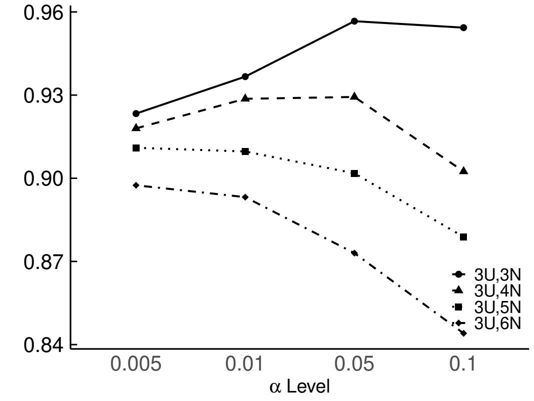

(b) Precision

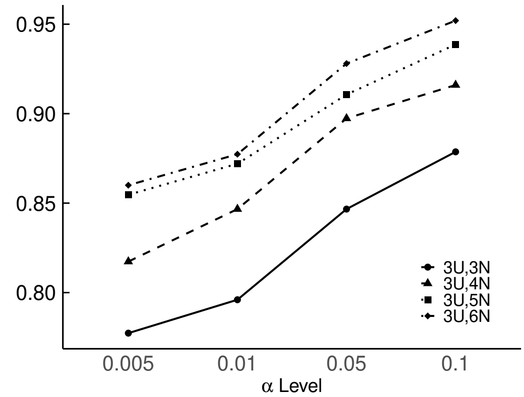

(c) Recall

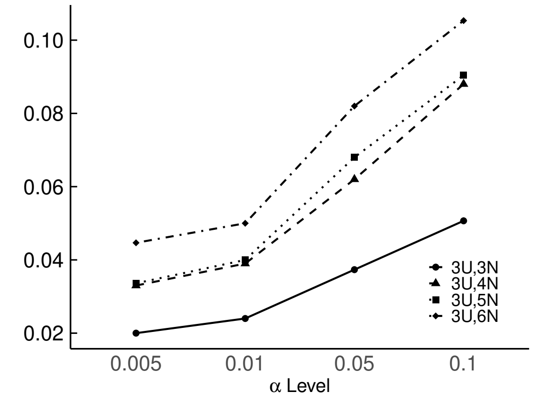

(d) Type I errors

Web Figure 3. Sensitivity analysis of model performance across varying α levels (0.005 to 0.1) under four simulation scenarios of different **Z** using two-step method with MI: 3U, 3N (3 useful **Z**, 3 noise **Z**); 3U, 4N (3 useful **Z**, 4 noise **Z**); 3U, 5N (3 useful **Z**, 5 noise **Z**); and 3U, 6N (3 useful **Z**, 6 noise **Z**). Each subfigure shows the metric trends: (a) F_1, (b) Precision, (c) Recall, and (d) Type I error. The definition of the four evaluation metrics are introduced in section 3.2. Distinct line types and symbols are used to represent different simulation settings.

E Names and definitions of variables used in the case study in Section 4

Variable names	Definition
chooseprobeaccavg	Generalization accuracy on the Concurrent Discrimination and Transfer Task
age	age in years
gender	gender
edu	education in years
restingsysBP	Resting systolic blood pressure in mmHg
restingHR	Resting heart rate in beats per minute
psqi_sleepquality	Self-reported sleep quality
BMI	Body Mass Index
VO_2max	Estimated Aerobic Fitness
ACS_MEDIAN_HH_INC	Median household income
ACS_MEDIAN_RENT	Median gross rent
ACS_MEDIAN_YEAR_BUILT	Median year structure built of housing units
ACS_PCT_DISABLE	Percentage of population with a disability
ACS_PCT_EMPLOYED	Percentage of civilian labor force that is employed (ages 16 and over)
ACS_PCT_HH_FOOD_STMP_BLW_POV	Percentage of households receiving food stamps/SNAP with income below the poverty level
ACS_PCT_HS_GRADUATE	Percentage of population with only high school diploma (ages 25 and over)
ACS_PCT_PUBLIC_OTHER	Percentage of population with other public-only healthcc insurance combinations
ACS_PCT_RENTER_HU	Percentage of occupied housing units: rented
ACS_PCT_UNINSURED	Percentage of population with no health insurance coverage

F Individual-level coefficients estimates (95% confidence intervals) from the unified and two-step model

Variables	**Unified model**		**Two-step model**	
	Zip code level	City level	Zip code level	City level
BMI	0.10 (-0.08, 0.08)	0.10 (-0.02, 0.22)	0.11 (-0.02, 0.23)	0.10 (-0.02, 0.23)
edu	-	0.15 (0.04, 0.26)	-	0.15 (0.04, 0.25)
VO_2max	0.20 (0.05, 0.35)	0.19 (0.04, 0.33)	0.20 (0.05, 0.35)	0.19 (0.04, 0.34)
